# ABC transporter inhibition by beauvericin partially overcomes drug resistance in *Leishmania tropica*

**DOI:** 10.1128/aac.01368-23

**Published:** 2024-04-04

**Authors:** Charbel Al Khoury, Sergio Thoumi, Sima Tokajian, Aia Sinno, Georges Nemer, Mark El Beyrouthy, Kelven Rahy

**Affiliations:** 1 Department of Natural Sciences, School of Arts and Sciences, Lebanese American University, Beirut, Lebanon; 2 Department of Computer Science and Mathematics, Lebanese American University, Beirut, Lebanon; 3 Department of Natural Sciences, School of Arts and Sciences, Lebanese American University, Byblos, Lebanon; 4 Division of Genomics and Translational Biomedicine, College of Health and Life Sciences, Hamad Bin Khalifa University, Doha, Qatar; 5 Department of Agriculture and Food Engineering, Holy Spirit University of Kaslik, Jounieh, Lebanon; 6 Gilbert and Rose-Marie Chagoury School of Medicine, Lebanese American University, Byblos, Lebanon; The Children's Hospital of Philadelphia, Philadelphia, Pennsylvania, USA

**Keywords:** *Leishmania tropica*, leishmaniasis, drug resistance, ABC transporter

## Abstract

Leishmaniasis is a neglected tropical disease infecting the world’s poorest populations. Miltefosine (ML) remains the primary oral drug against the cutaneous form of leishmaniasis. The ATP-binding cassette (ABC) transporters are key players in the xenobiotic efflux, and their inhibition could enhance the therapeutic index. In this study, the ability of beauvericin (BEA) to overcome ABC transporter-mediated resistance of *Leishmania tropica* to ML was assessed. In addition, the transcription profile of genes involved in resistance acquisition to ML was inspected. Finally, we explored the efflux mechanism of the drug and inhibitor. The efficacy of ML against all developmental stages of *L. tropica* in the presence or absence of BEA was evaluated using an absolute quantification assay. The expression of resistance genes was evaluated, comparing susceptible and resistant strains. Finally, the mechanisms governing the interaction between the ABC transporter and its ligands were elucidated using molecular docking and dynamic simulation. Relative quantification showed that the expression of the ABCG sub-family is mostly modulated by ML. In this study, we used BEA to impede resistance of *Leishmania tropica*. The IC_50_ values, following BEA treatment, were significantly reduced from 30.83, 48.17, and 16.83 µM using ML to 8.14, 11.1, and 7.18 µM when using a combinatorial treatment (ML + BEA) against promastigotes, axenic amastigotes, and intracellular amastigotes, respectively. We also demonstrated a favorable BEA-binding enthalpy to *L. tropica* ABC transporter compared to ML. Our study revealed that BEA partially reverses the resistance development of *L. tropica* to ML by blocking the alternate ATP hydrolysis cycle.

## INTRODUCTION

Leishmaniasis, a vector-borne zoonotic infection, is widely associated with poverty and predominantly occurs in the most impoverished parts of developing countries (1). The epidemiology of leishmaniasis has undergone changes in recent years, with the disease becoming endemic in numerous regions, including tropical and sub-tropical areas of Africa, America, and Asia, as well as in rural and urban areas in southern Europe (2). The disease is caused by *Leishmania*, a genus of trypanosomes, and spreads through the bite of a phlebotomine sandfly (3). Three different clinical forms of leishmaniasis were documented: cutaneous leishmaniasis, which causes ulcers on the skin; visceral leishmaniasis, also known as kala-azar, which affects several internal organs such as the spleen, liver, and bone marrow; and mucocutaneous leishmaniasis, a subset of cutaneous leishmaniasis that can occur several months after the skin ulcers have healed (4). Clinical manifestations of the disease largely depend on the species of the infecting protozoan. Each year, an estimated range of 600,000–1 million cases of cutaneous leishmaniasis and 50,000–90,000 new cases of visceral leishmaniasis are reported (5). In the realm of parasitic diseases, leishmaniasis ranks as the second leading cause of human mortality, following closely behind malaria (6). Climate change has exacerbated the ecological risk of host exposure to leishmaniasis. Environmental changes have had a positive impact on the survival and distribution of the sandfly vector. Concomitantly, a larger geographical region is currently inhabited by the parasite and its vector (7).

During its life cycle, the parasite alternates between a phlebotomine sandfly (vector) and a mammal (host) (8). The extracellular form of the parasite, known as promastigote, is located in the midgut of the insect vector and is regurgitated during a blood meal. This flagellated stage is found to harbor parasite-associated antigens that are recognized by surface receptors of host monocytes (9). The invading pathogen is then phagocytized by macrophages, where they reside within the phagolysosomal vacuole. Interestingly, the parasite dwells inside the host’s mononuclear phagocyte system of the host and differentiates into amastigote, the intracellular stage of the parasite (10). Few drug companies are interested in developing new therapeutic options for neglected diseases. Antimonials are the first-line treatment for leishmaniasis, but the emergence of resistance and toxicity has become a major concern (11). Miltefosine (ML) is the only approved oral treatment for leishmaniasis. ML, a phosphatidylcholine analog, has one of the highest therapeutic indices against leishmaniasis (12). It induces apoptotic-like cell death in *Leishmania* by interfering with signaling pathways and cell membrane synthesis (12). However, there is a rapid increase in ML-resistant *Leishmania* spp., endangering its efficacy and potency (13). To address the growing problem of resistance, higher doses of ML were recommended to overcome it (14), which, unfortunately, led to a doubling of the relapse rate. Resistance to ML in *Leishmania* can be associated with complex changes in drug transport mechanisms, including both decreased drug influx and increased drug efflux (15). The miltefosine transporter (MLT), operating as a flippase enzyme, in conjunction with its associated protein, Ros3, is demonstrated to play a pivotal role in the emergence of ML resistance by exerting control over the translocation of ML across the parasitic membrane (16). The loss of these proteins results in a diminished intracellular concentration of ML, thereby attenuating its therapeutic efficacy (16).

Moreover, ATP-binding cassette (ABC) transporters, which function as transmembrane ATP-powered pumps, play a pivotal role in *Leishmania* by facilitating the transport of various molecules, with some closely linked to drug resistance mechanisms (17). They are composed of two highly conserved cytoplasmic nucleotide-binding domains (NBDs) and two diverse transporter domains, also known as transmembrane domains (TMDs). Together, these elements form the “N-TMD1-NBD1-TMD2-NBD2-C” topology (18). This ubiquitous superfamily of integral membrane proteins uses the energy provided by ATP hydrolysis in their nucleotide-binding sites (NBS) to translocate xenobiotic compounds after binding to the drug-binding site (DBS). ABC transporters mediate resistance in *Leishmania* by the efflux of anti-parasitic agents (19). One way to overpower ABC transporter-controlled multidrug resistance (MDR) is to search for potent ABC transporter inhibitors capable of resensitizing the parasite to anti-microbial agents (20). The first attempts at this approach were recorded by Pérez-Victoria et al. (19), who showed a flavonoid derivative with a high affinity for the cytosolic NBD overcomes the ABC transporter-mediated ML resistance in *Leishmania*. Beauvericin (BEA), a depsipeptide belonging to the enniatin family, is produced by the necrotrophic fungus *Beauveria bassiana* (Balsamo) Vuillemin (Hypocreales: Cordycipitaceae). It is a fungal secondary metabolite and a promising candidate to abrogate drug resistance (21). BEA enhanced the therapeutic index of ketoconazole and was used in the treatment of MDR strains of *Candida albicans* by blocking ABC transporters (22). Similarly, our group recently demonstrated that BEA can potentiate the activity of sub-efficient pesticides against *Tetranychus urticae* Koch (Trombidiformes: Tetranychidae), the most resistant pest species (23). In this study, we tested the dynamics of ML transport, including permeation across the cellular membrane and its expulsion from the intracellular milieu in resistant and susceptible strains of *Leishmania tropica*. Moreover, we assessed BEA as a potential therapeutic agent against *L. tropica* through targeting ABC transporters. We also determined the transcription profile of ABC transporters (and other genes) involved in drug resistance and predicted the mechanism of ABC transporter inhibition through molecular docking and dynamic simulation.

## MATERIALS AND METHODS

### Parasite culture and maintenance

*Leishmania tropica* LT2 (strain designation: MHOM/LB/2015/IK) was isolated in 2014 from skin punch biopsies collected at the American University of Beirut Medical Center with detailed patient annotations, as previously described (approval reference #PALM I.K.01) (24). *Leishmania* promastigotes were cultured in RPMI-1640 medium (Sigma-Aldrich #R8758), 20% heat-inactivated fetal bovine serum (FBS) (#10500064, Thermo), and 1% penicillin-streptomycin (#17–602E, Lonza). Cultures were incubated at 25°C in a 5% CO_2_ incubator. *Leishmania* promastigotes were used to initialize axenic amastigotes, as previously described (25). THP-1 cells were maintained in RPMI-1640 (with 10% FBS and 1% penicillin-streptomycin) at 37°C in a 5% CO_2_ incubator. The cells were induced to become adherent, adopting a mature macrophage-like phenotype, in TPP plates (#92006) by the addition of 50-ng/mL phorbol 12-myristate 7-acetate from a stock of 1 mg/mL (#BP685–1, Fisher). Plates were incubated at 37°C in a 5% CO_2_ incubator overnight to allow complete differentiation of the cells. Next, cells were washed three times with phosphate-buffered saline (PBS) Sigma-Aldrich (D8662) and stimulated with lipopolysaccharide (#TLRL-EBLPS, Invivogen) at a concentration of 1 ng/mL for 4 h. To culture intracellular amastigotes, 1 × 10^6^ cells/well were seeded into a 96-well plate in 100 µL of RPMI-1640 medium supplemented with 20% FBS and 1% penicillin-streptomycin. Macrophages were infected 24 h later with metacyclic promastigotes at a 1:10 (macrophage:promastigote) multiplicity of infection and placed overnight in a 37°C, 5% CO_2_ incubator. The cells were then washed twice with PBS to remove non-internalized promastigotes. Finally, intracellular amastigotes were incubated for an additional 24 h to establish the infection.

### Resistance selection

*L. tropica*, the susceptible strain (LS-LT2), was maintained without drug exposure. Cell viability was measured by the absolute quantification of gene expression using reverse transcription quantitative PCR (RT-qPCR) (26). This approach was found to be more sensitive and time efficient compared to the other conventional methods, including microscopic counts, fluorometric assays, and colorimetric assays (26). In brief, total RNA was extracted from both treated and non-treated control cells using the RNeasy mini kit (Qiagen, Hilden, Germany), following the manufacturer’s instructions. Subsequently, 2 µg of RNA was reverse transcribed into cDNA using the Revert Aid First cDNA Synthesis Kit (#K1622, Thermo Scientific). SYBR Green 2× (Sigma-Aldrich) was used to quantify the expression of kDNA minicircles and *GAPDH*, which serve as markers for the parasites and macrophages, respectively. Next, double-stranded DNA purified from conventional PCR reactions was used as standard. The quantification of the input target sequence was achieved by plotting the *C*_t_ values of the cells on the standard curves (26).

To obtain ML-resistant strains, LS-LT2 was exposed for 72 h to increasing ML concentrations (0–50 µM). At each round of selection, the drug concentration was adjusted to exert selection pressure, killing 50% of the parasite population. Every five rounds of selection, the efficacy of ML was evaluated against different developmental stages of *L. tropica*, and the IC_50_ was calculated for that round of selection. After 15 rounds of selection of induced selection, the strain resistant to ML was designated as MLR-LT2. Resistance ratios (RRs) were calculated by dividing the IC_50_ value of the round of selection under investigation by the IC_50_ value of the parental strain LS-LT2. This experiment was conducted three times in triplicate.

### *In vitro* resistance study: long-term exposure of axenic amastigotes

Promastigotes and axenic amastigotes were cultured at a concentration of 1 × 10^6^ cells/mL in 10 mL of supplemented RPMI-1640 medium. Cells were exposed to successive doses of ML to exert selection pressure, killing 50% of the initial population. After 72 h post-inoculation, parasites were transferred to a fresh medium and left for another 72 h. The number of cells was quantified (10^6^ cells/mL in 10 mL), transferred into a new flask, and subjected to the drug again.

### *In vitro* resistance study: long-term exposure of intracellular amastigotes

Intracellular amastigotes were seeded in 96-well plates and exposed for 72 h to the amount of ML required to kill 50% of the test parasites. The plates were then washed three times with PBS. For parasite rescue and transformation, PBS was removed, and surviving cells were treated with 20 µL of RMPI-1640 supplemented with 5% sodium dodecyl sulfate (SDS) (Bio-Rad 1610301) (27). After shaking for 30 s, 180 µL of RPMI (supplemented with 10% FBS) was added to each well. This step allows the controlled lysis of infected macrophages with minimum loss of viability for the rescued parasites (27). To fully transform intracellular amastigotes into promastigotes, the plates were incubated at 27°C for 48 h. After detecting promastigotes, further expansion was carried out in a 25-mL flask. The metacyclic promastigotes were then used for the next infection round of macrophages. After phagocytosis, the intracellular amastigotes were once again exposed to the drug. To evaluate the infectivity of the rescued promastigotes, we monitored the infection rate by staining macrophages with Giemsa at intervals of every five rounds of selection. A minimum of 500 cells were assessed across 10 separate microscopic fields to determine the total number of infected macrophages.

### Resistance inhibition assay

The ability of BEA to restore responsiveness to treatment in ML-resistant *L. tropica* (MLR-LT2) was tested. To eliminate the possibility of false-positive results following combinatorial therapy, a pilot study was conducted to determine the sub-lethal doses of BEA against *L. tropica*. An exposure-response assay was performed by adding increasing BEA concentrations to *L. tropica* cultures. The highest dose of BEA that preserved the viability of *L. tropica* without any significant difference compared to the negative control (water) was found to be 0.01 µM. Verapamil was used as a positive control due to its previously demonstrated ability to restore the responsiveness of *Leishmania-*resistant strains (28, 29). The IC_50_ of ML against all developmental stages of MLR-LT2 was calculated as indicated above. Subsequently, test cells were exposed to the combination of ML and 0.01-µM BEA or ML and 15 µM of verapamil, while control cells received only the single ML dose. This experiment was repeated three times in triplicate.

### Gene expression profiling of resistance genes

Total RNA was extracted from different developmental stages using the RNeasy mini kit (Qiagen), following the manufacturer’s protocol. Comparative transcriptomics assessed the role of 15 genes (A*BCB2*, *ABCB4*, *ABCC3*, *ABCC7*, *ABCG4*, *ABCG6*, *ML-T*, *Ros3*, *CYP5122A1*, *HSP83*, *HSP60*, *HSP70*, *PRX1A*, *SAMS*, and *PCNA*) in the acquisition of resistance by *L. tropica* to ML (Table S1). Actin was chosen as a housekeeping gene for its demonstrated expression stability under various biological conditions (30). Total RNA from the susceptible (LS-LT2) and resistant (MLR-LT2) strains of *L. tropica* served as calibrator and test values, respectively. The mean *C*_t_ numbers collected from non-treated parasites were considered as control. Nucleic acids were quantified using the NanoDrop at 260 and 280 nm (NanoDrop Technologies, Wilmington, DE, USA). RNA quality was assessed by 1% agarose gel electrophoresis. Following the manufacturer’s instructions, 2 µg of total RNA was reverse transcribed into cDNA using iScript (Bio-Rad, USA). PCR reactions were performed as previously described (23). PCR efficiency was estimated using standard curves constructed by plotting *C*_t_ values versus the log of concentrations (10-fold serial dilutions) and calculated as *E* = 10^(−1/slope)^. Primer specificity was confirmed by obtaining a single band of the expected size using gel electrophoresis and a high single peak using RT-qPCR melting curve analysis. Moreover, the amplicon sequencing was performed using either the forward or the reverse primers on an ABI3500 instrument (31) (supplemental material). A no-template control was generated to detect primer dimerization and unspecific amplification. The RT-qPCR experiment was repeated three times in triplicate, and the mean values were used for the gene expression analysis. Fold differences in gene expression were calculated using the Livak method (2^−ΔΔCq^) (32).

### Production of non-functional ABC transporter

In consideration of its prominent upregulation following exposure to ML, *ABCG6* was chosen for a detailed examination to elucidate its role in the acquisition of resistance in *L. tropica*. Employing site-directed mutagenesis, as outlined in previous studies (33), ABCG6 was isolated from *L. tropica* genomic DNA through PCR amplification with the primers 5′-TCACTTTCCCTCAGTGGACC-3’ and 5’-AACAGCTACGCTCTCTGCC-3′. Sequencing of the amplicon was carried out using either the forward or reverse primers on an ABI3500 instrument (Thermo Fisher Scientific, USA) to confirm its identity (31). To generate parasites overexpressing non-functional ABCG6, a targeted disruption was introduced by mutating the Walker A motif in the ATP binding domain, specifically substituting lysine 403 with methionine (K403M), utilizing the QuikChange XL Site-Directed Mutagenesis kit (Stratagene, La Jolla, CA, USA) according to the manufacturer’s protocol. The resulting modified gene, *ABCG6*^K403M^, was cloned into the *Leishmania* expression vector pUCNeoPlus (34). Transfection of parasites and subsequent selection for G-418 resistance followed established protocols (35). The strain overexpressing non-functional ABCG6 was designated LS-LT2^K/M^, and confirmation of gene overexpression was conducted through RT-qPCR, as detailed above.

### The internalization of labeled phosphocholine and ML

Phosphocholine and ML internalization experiments were conducted using rescued amastigotes of LS-LT2 and MLR-LT2 following the protocol outlined (16). Amastigotes of the parasite were immersed in a specialized solution comprising HEPES-NaCl buffer (composed of 21-mM HEPES, 137-mM NaCl, 5-mM KCl, 0.7-mM NaH_2_PO_4_, 6-mM glucose, pH 7.05). To prevent phospholipid degradation, this pre-treatment, lasting 15 min, involved the addition of 0.3% bovine serum albumin (BSA) and 500-µM phenylmethylsulfonyl fluoride (PMSF) (Sigma-Aldrich). Subsequently, either 10-µM 2-(4,4-difluoro-5-methyl-4-bora-3a,4a-diaza-s-indacene-3-dodecanoyl)−1-hexadecanoyl-sn-glycero-3-phosphocholine)] (BODIPY-PC) (Invitrogen) or 1-µM BODIPY-labeled ML [[11-(4′,4′-difluoro-6′-ethyl-1′,3′,5′,7′-tetramethyl-4′-bora3′a,4′a-diaza-s-indacen-2′-yl)-undecylphosphocholine] (ML-EtBDP)] (36) was introduced. Following this, the parasites underwent further incubation for either 1 h or 5 min, maintaining a temperature of 25°C. To eliminate any residual labeled molecules external to the cells, the parasites underwent a rigorous washing process involving three rounds of rinsing with ice-cold HEPES-NaCl containing 0.3% BSA. Finally, the parasites were suspended in PBS for analysis through flow cytometry, employing the Guava EasyCyte HT. Each sample was evaluated based on 20,000 events. This experiment was conducted three times in triplicate.

### Quantification of residual ML

Rescued amastigotes of LS-LT2, LS-LT2^K/M^, and MLR-LT2 were subjected to an incubation period lasting 5 min with 1-µM ML-EtBDP, all within a medium comprising HEPES-NaCl buffer as previously suggested (16). This medium was further supplemented with 0.3% BSA and 500-µM PMSF to ensure the integrity of phospholipids. Following this brief incubation, parasites underwent a series of rinses with HEPES-NaCl 0.3% BSA. After these preparations, 10 × 10^6^ parasites were resuspended in a solution of 1-mg/mL PBS-glucose for the quantification of ML-EtBDP uptake. Subsequently, at time intervals of 1, 2, 7, and 24 h, the parasites were pelleted, and the supernatant was collected. Additional washing steps with HEPES-NaCl 0.3% BSA were carried out to eliminate any remnants of non-internalized molecules. The quantification of residual fluorescence retained within the parasites was accomplished using flow cytometry, facilitated by the Guava EasyCyte HT. A comprehensive analysis of 20,000 events was conducted for each assessment. The outcomes were expressed as the proportion of fluorescence remaining inside the parasites in comparison to the initial fluorescence levels following the initial uptake phase. This experiment was conducted three times in triplicate.

### Preparation of plasma membranes and ATPase activity assay

The plasma membrane and the ATPase activities of the isolate parasite membranes were performed exactly as previously described (37). Rescued amastigotes (5 × 10^8^ cells/mL) of LS-LT2, LS-LT2^K/M^, and MLR-LT2 were harvested via centrifugation at 500 × *g* for a duration of 3 min and subjected to triplicate aqueous wash steps utilizing chilled PBS. Mechanically induced cellular disruption was performed by abrasion of the leishmanial suspension with spherical glass beads (diameter 0.5 mm) by constant agitation on an ice bath for 8 min. Subsequently, a 25-mL aliquot of homogenization buffer (10-mM HEPES, pH 7.4, 400-mM mannitol, 10-mM KCl, 1-mM magnesium acetate, 1-mM PPMSF, 10-µM, and 1-µM pepstatin A) was added to the mixture. Unruptured cells and macroscopic debris were eliminated by slow-speed centrifugation at 1,000 × *g* for 20 min at 4°C. To pellet organelles and enrich membranes, the resultant supernatant was sequentially centrifuged at increasing speeds of 5,000 × *g*, 16,000 × *g*, and 105,000 × *g* for 20, 40, and 60 min, respectively. The sedimented pellet following the final ultracentrifugation was gently resuspended in buffer comprising 150 mM (2-mM MgCl_2_, 1-mM dithiothreitol, and 75-mM HEPES, pH 7.4), then layered on a continuous density gradient of 18% Percoll in 0.25-M sucrose and 12-mM Tris-HCl, pH 7.4. Subsequent to centrifugation at 40,000 × *g* for 1 h, the light density fractions enriched for plasma vesicles were recovered and maintained at −80°C after reisolation by sedimentation and resuspension in 250-mM sucrose at concentrations of 20-mg/mL protein. For the ATPase assay protocol, the standard reaction mixture consisted of 20-mM HEPES-Tris, pH 7.0, 10-mM MgCl_2_, 5-mM ATP, and ML or ML + BEA. The enzymatic hydrolysis of ATP was quantified by monitoring the liberation of inorganic phosphate from [γ^32^P]-ATP (10^4^ Bq/nmol ATP) as previously documented (38). The reaction was initiated by the addition of plasma vesicles to achieve a final protein concentration of 0.5 mg/mL. After 1 h, the reaction was terminated by the addition of 0.5-mL activated charcoal suspended in 0.1-N HCl. The released [^32^P] Pi was assessed in a 0.2-mL fraction of the supernatant obtained after centrifugation of the charcoal mixture at 1,500 × g for 15 min at 4°C and supplementation with 10-mL scintillation fluid containing 2-g PPO (2,5-diphenyloxazole)/L of toluene. This experiment was repeated three times, with different membrane preparations, in triplicate.

### Building the simulation system

ABCG6 was selected for further molecular docking and dynamic simulation studies due to the prominent overexpression of *ABCG6* in response to ML, as revealed through our comprehensive monitoring of differential expression among multiple ABC transporters. Homology-based structure of ABCG6 was constructed using the Alignment Mode algorithm of SWISSMODEL (39–41). The inward-facing multidrug transporter P-glycoprotein from *Caenorhabditis elegans* (PDB ID: 4F4C) was selected as a template based on the GlobalModel Quality Estimate (Table S2). The modeled structure of ABCG6 was refined by optimizing the hydrogen bonding network and atomic-level energy minimization (42). The best model was selected based on the MolProbity score, which assesses the structural quality of the entire structure (43). The folding of the linker was assessed through secondary structure prediction tools using PSIPRED, v.3 (44), and PROF (45) which showed the absence of regions with secondary structures in the linker (data not shown). The incorporation of a lipid bilayer is crucial for understanding the extrusion mechanism of chemotherapeutics. To shed light on the receptor-ligand interactions, the ABC transporter was embedded in a simplified membrane lacking cholesterol. It has been demonstrated that the presence of 10% cholesterol does not affect the efflux mechanism and pathway of human P-glycoprotein, as long as xenobiotics do not accumulate in the cholesterol-rich regions of the membrane (46, 47). The homology-based structure was inserted into a 1-palmitoyl-2-oleoylphosphatidylcholine (POPC) bilayer using the Lipid Builder of CHARMM-GUI (http://www.charmm-gui.org/) (48). Parameterization was based on previous simulations (46, 47). In summary, the lipid bilayer had dimensions of 13.84 × 14.44 nm (corresponding to a total of 379 POPC) in the xy-plane, with 190 and 189 POPC lipid molecules in the upper and the lower layers, respectively (46, 47).

### Molecular docking

The crystal structure of BEA (T3D3740) was downloaded from the Toxin and Toxin Target Database (www.t3db.ca/toxins), while those of the ML (PubChem ID: 3599) and remaining drugs were obtained from PubChem (https://pubchem.ncbi.nlm.nih.gov). Verapamil (PubChem ID: 2520), cyclic-tris-(S)-valineselenazole “QZ59RRR” (PubChem ID: 25195366), and cyclic-tris-(R)-valineselenazole “QZ59SSS” (PubChem ID: 25195367) were employed as positive controls due to their known role in inhibiting ABC transporters (43, 47). The ligands were loaded in .sdf format and automatically converted into a three-dimensional structure during the docking process. The resulting transmembrane protein systems (ABC transporters, ABCG6, plus the bilayer) were imported into Molsoft.icm-pro, v.3.9–1b (49), and then transformed into ICM (internal coordinate mechanics) objects. This transformation involved deleting water molecules, optimizing hydrogens, and processing amino acids including histidine, proline, asparagine, glycine, and cysteine. Missing side chains were also hidden. The internal cavity of the ABC transporter, delimited by TMD, was considered the DBS (50). The energy map was defined within a ~6,000-Å^3^ pocket, taking into account hydrogen bonding potential, van der Waals potential with carbon-, sulfur- and hydrogen-like probes, hydrophobic potential, and electrostatic potential. Conformational exploration was carried out using the biased probability Monte Carlo system (51). Docking was performed with static target crystal structures and repeated three times. The simulation length (thoroughness) was set as 30, and ligand conformations were ranked using the ICM score (49).

### Molecular dynamics

We employed molecular dynamics (MD) to investigate the impact of the substrate (ML) and inhibitor (BEA) on the conformation of the ABC transporter (ABCG6). Our study involved 100-ns simulations with the apo ABC transporter in the absence of a ligand. Subsequently, we analyzed the ML-bound ABC (MBA) and BEA-bound ABC (BBA) ABC transporters. MD simulation and the binding free energy calculations were conducted as previously described (52). In brief, MD simulations were executed on the apo protein and protein-ligand complexes using GROningen MAchine for Chemical Simulations (GROMACS v.2022.4) with the GPUacceleration (53). The topologies of proteins were generated using the CHARMM36 force field in GROMACS format (. gro), which was obtained from the Mackerell lab website (http://mackerell.umaryland.edu/charmm_ff.shtml#gromacs). CHARMM General Force Field CGENFF was converted into.gro format using the script available at the Mackerell laboratory website (cgenff_charmm2gmx_py3_nx1.py) for the ligands. The complexes were placed in a dodecahedral unit cell, and the SPC (Simple Point Charge) model was used for water molecules. To neutralize all systems, ions were added to the simulation box using the “gmx genion” script. The systems underwent energy minimization (>10 kJ/mol) (54), followed by simulation at NVT [simulation ensemble in which the number of particles (N), volume (V), and temperature (T) are kept constant] (2 ns) and NPT [simulation ensemble in which the number of particles (N), pressure (P), and temperature (T) are kept constant] (8 ns) ensembles. Long-range electrostatic interactions were handled using the particle mesh Ewald method with a 12-Å cut-off and 12-Å Fourier spacing (55). Bond constraints for all heavy atoms were applied using the LINCS (LINear Constraint Solver) algorithm (56). The systems were finally subjected to MD simulations for 100 ns using the leapfrog method. Various parameters, including root mean square fluctuation, root mean square deviation (RMSD), radius of gyration (Rg), and peptide-peptide hydrogen bonds (H bonds) were calculated from the generated MD simulation trajectories using GROMACS scripts. For the calculation of binding free energy between the ABC transporter and different substrates, we employed the molecular mechanics Poisson-Boltzmann surface area (MM/PBSA) methods (57) using the g_mmpbsa tool of GROMACS (58), as previously described (52). The average of three independent trajectories was used for analysis.

### Analysis of the nucleotide-binding domains

Further analyses were carried out to elucidate the effect of the ligand characteristic (substrate or inhibitor) on ATP hydrolysis of ABCG6. Firstly, we evaluated key distances to monitor the separation of NBDs throughout the trajectory. Specifically, we measured the Cα-Cα distances between the N-terminal Walker A motif (Lys403) and the C-terminal signature sequence (Ser1166) (referred to as “site 1”) and between the C-terminal walker A motif (Lys1067) and the N-terminal signature sequence (Ser502) (referred to as “site 2”). Next, we annotated the NBS based on sequence conservation and interactions observed in co-crystallized structures between ATP and related ABC transporters (four reference structures) (Table S3). Subsequently, we compared the geometry of NBS sites in MBA and BBA through superimposition with those of ABC transporters co-crystallized with ATP (Table S3). Finally, we assessed the competency of these sites by docking ATP using Molsoft.icm-pro, as mentioned above.

### Statistical analysis

IC_50_ values and their standard errors were calculated using probit regression in Statistics for Windows, v.25.0 (SPSS). To assess the ability of BEA to reverse ML-acquired resistance, monitor the influx and efflux of ML, investigate the ATPase activity, and analyze the relative expression data, we employed a statistical comparison test of means through one-way analysis of variance. Additionally, the Tukey test was used to separate means, with a significance threshold set at 5%.

## RESULTS

### Induction of resistance in *L. tropica*

The *in vitro* efficacy of ML against different stages of *L. tropica* is presented in Table 1. Prior to the selection assay, the IC_50_ values of ML against promastigotes, axenic amastigotes, and intracellular amastigotes were 1.5, 1.1, and 2.6 µM, respectively (Table 1). Notably, intracellular amastigotes exhibited less resistance compared to promastigotes and axenic amastigotes. However, after 15 rounds of selection, the IC_50_ values for promastigotes, axenic amastigotes, and intracellular amastigotes increased to 30.83, 48.17, and 16.9 µM, respectively. This resulted in RR ranging from 6.5- to 43.73-fold, indicating significant resistance in all life stages of *L. tropica* to ML (Table 1). A significant difference [degree of freedom (df) = 1; *F* statistics (*F*) = 41, 55; “probability value” (*P*) < 0.05] was detected in the infectivity rates of the rescued promastigotes following one round of ML-selection pressure (97.5% ± 4.2), compared to subsequent rounds, namely, 5 (93.4% ± 3.9), 10 (87.4% ± 5.3), and 15 (79.7 % ±4.4) rounds of ML-selection pressure.

**TABLE 1:** Efficacy of ML against different developmental stages of *L. tropica* with calculated resistant ratios

	Promastigotes		Axenic amastigotes		Intracellular amastigotes	
Population	IC_50_ (µM) ± SE	RR^*a*^ ± SE	IC_50_ (µM) ± SE	RR ± SE	IC_50_ (µM) ± SE	RR ± SE
**1**	1.50 ± 0.10	1.00 ± 0.00	1.10 ± 0.03	1.00 ± 0.00	2.64 ± 0.0	1.00 ± 0.00
**5**	3.51 ± 0.21	2.33 ± 0.13	2.71 ± 0.22	2.47 ± 0.23	3.18 ± 0.0	1.23 ± 0.08
**10**	13.68 ± 0.43	9.20 ± 0.99	20.83 ± 0.38	20.83 ± 0.36	6.72 ± 0.33	2.61 ± 0.05
**15**	30.83 ± 0.72	20.34 ± 1.00	48.17 ± 0.53	43.73 ± 1.62	16.93 ± 0.36	6.56 ± 0.53

^*a*^ Resistance ratios (RRs) were calculated by dividing the IC_50_ value of the round of selection under investigation by the IC_50_ value of the parental LS-LT2 strain.

### Assessing BEA’s ability to counteract ML-acquired resistance

In the conducted pilot study, we did not observe a difference between the developmental stages of *Leishmania* exposed to water or 0.01 µM of BEA *in vitro* (data not shown). We evaluated the efficacy of ML alone and in combination with BEA or verapamil (a known P-glycoprotein inhibitor) against *L. tropica* MLR-LT2 *in vitro*. With the combinatorial treatments, the activity of ML improved significantly compared to the control (ML alone). Survival of promastigotes (df = 2; *F* = 75, 73; *P* < 0.05), axenic amastigotes (df = 2; *F* = 98, 445; *P* < 0.05), and intracellular amastigotes (df = 2; *F* = 51, 91; *P* < 0.05) exposed to ML-BEA combination was significantly lower when compared to ML-verapamil as well as the single-ML dose application (Table 2).

**TABLE 2:** Susceptibility of *L. tropica* resistant strain (MLR-LT2) exposed to miltefosine alone and in combination with beauvericin or verapamil

	Promastigotes		Axenic amastigotes		Intracellular amastigotes	
	IC_50_ (µM) ± SE	RR ± SE	IC_50_ (µM) ± SE	RR ± SE	IC_50_ (µM) ± SE	RR ± SE
Miltefosine	30.83 ± 0.78	20.34 ± 1.00	48.17 ± 0.51	43.73 ± 1.61	16.93 ± 0.56	6.52 ± 0.38
Miltefosine + beauvericin	8.10 ± 0.85	5.42 ± 0.62	10.94 ± 0.66	9.93 ± 0.92	7.215 ± 0.35	2.774 ± 0.28
Miltefosine + verapamil	27.21 ± 1.24	18.14 ± 1.16	41.97 ± 1.47	38.15 ± 1.20	15.63 ± 0.67	6.00 ± 0.35

### Differential gene expression

**Fig 1:**
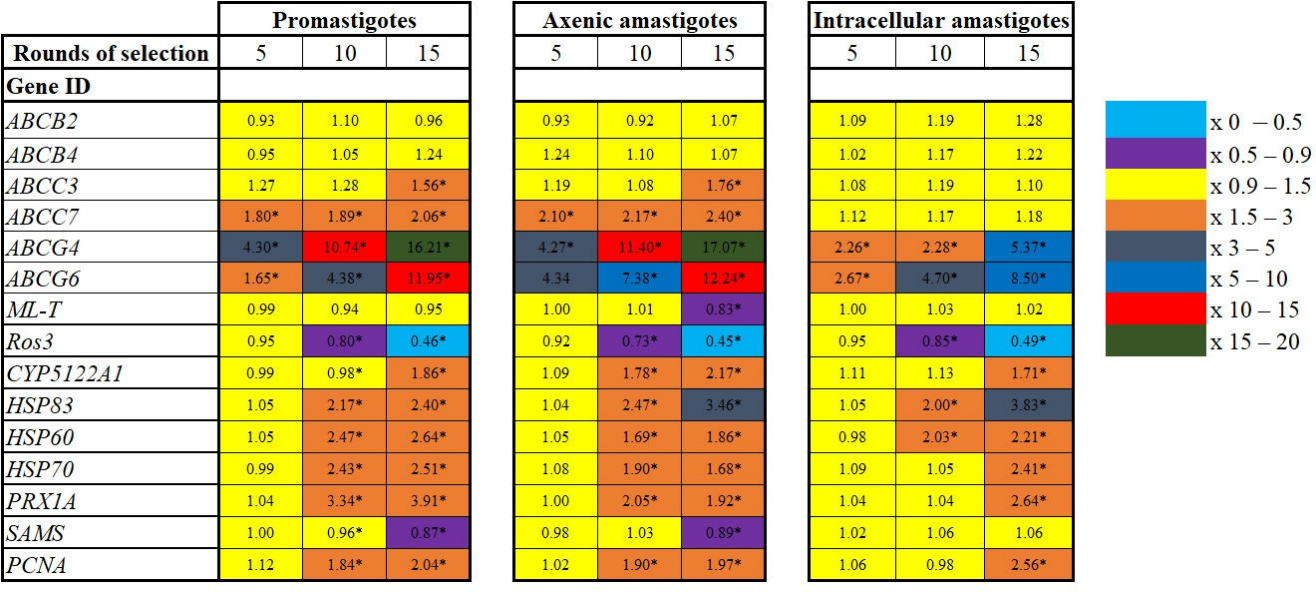
Transcription profiles of genes involved in resistance development, including ABC transporter genes (from the ABCB, ABCC, and ABCG sub-families), MLtransport complex (ML-T and Ros3), heat shock proteins (HSP83, HSP60, and HSP70), S-adenosylmethionine, cell nuclear antigen, cytosolic peroxiredoxins after the resistance selection of promastigotes, axenic amastigotes, and intracellular amastigotes to miltefosine. Transcription levels are shown as mean fold transcription of the *L. tropica* resistant strain (MLR-LT2) relative to the laboratory susceptible strain (LS-LT2). Asterisks indicate significant differences (one-way analysis of variance model, *P* ≤ 0.05).

The differential expression of six ABC transporter genes (from the ABCB, ABCC, and ABCG sub-families) across all developmental stages of *L. tropica* was analyzed. The tested ABCB genes, *ABCB2* and *ABCB4* encoding for multidrug resistance 2 (MDR2) and MDR1, respectively, demonstrated stable expression in all life stages following induced selection (Fig. 1). Strikingly, *ABCC3* that encodes for the multidrug-resistant protein A or P-glycoprotein A showed negligible overexpression in the promastigotes and axenic amastigote forms of *L. tropica*. Notably, *ABCC7*, encoding for pentamidine resistance protein 1, exhibited significant overexpression in the extracellular stages of the parasite, while no significant difference was detected in intracellular amastigotes. *ABCG4* and *ABCG6* were classified as “generalist genes” as they demonstrated overexpression in all the stages after ML exposure (Fig. 1). Based on the number of overexpressed genes and the average expression rate, transcription of the ABC transporters followed this pattern: axenic amastigotes > promastigotes > intracellular amastigotes (Fig. 1). Intriguingly, the *MLT* maintained a stable expression profile throughout all developmental stages of *L. tropica*, even over 15 rounds of stress selection. Conversely, the associated sub-part, *Ros3*, exhibited significant downregulation in resistant strains compared to their susceptible counterparts (Fig. 1). Moreover, within the spectrum of gene categories related to defense mechanisms, MLR-LT2 consistently displayed heightened transcription levels compared to LS-LT2. Notably, the *HSP83* gene manifested the most significant upregulation, while the *SAMS* gene exhibited the least pronounced alteration (Fig. 1).

### Uptake of ML-EtBDP and BODIPY-PC in *L. tropica* strains

**Fig 2:**
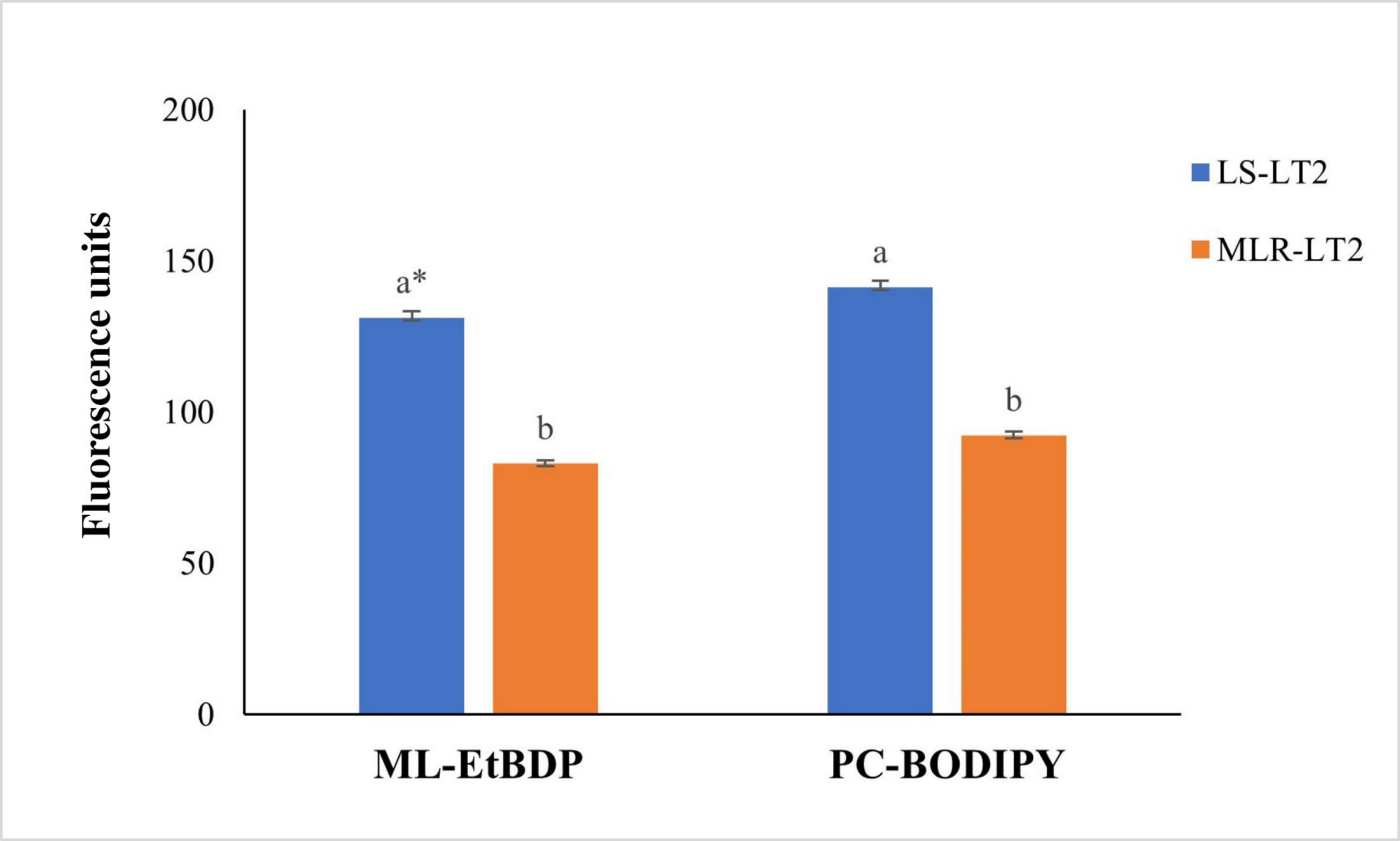
Incorporation of ML-EtBDP and PC-BODIPY by LS-LT2 and MLR-LT2 strains of *L. tropica*. Different *L. tropica* strains were cultured with labeled molecules, and the fluorescence intensity within the parasites was assessed using flow cytometry. One-way analysis of variance was utilized to evaluate the statistical significance of variations in fluorescence units among distinct *Leishmania* strains. *The values bearing different superscript letters within a column exhibit significant differences (*P* < 0.05).

We conducted flow cytometry to assess the uptake of ML-EtBDP and BODIPY-PC, examining the accumulation of these substances in both susceptible (LS-LT2) and resistant (MLR-LT2) *L. tropica* strains. Our investigation unveiled significant variations in the absorption of ML-EtBDP and BODIPY-PC between these distinct strains (Fig. 2). Additionally, an inverse association emerged between the quantities of ML-EtBDP and BODIPY-PC stored within the parasite and their susceptibility to ML. This correlation was substantiated by correlation coefficients of *r* = −0.975 (*P* < 0.05) for MLEtBDP and *r* = −0.964 (*P* < 0.05) for BODIPY-PC. In particular, LS-LT2, exhibiting an IC_50_ of 2.6 µM, displayed a heightened retention of both BODIPY-PC and ML-EtBDP. In contrast, MLR-LT2 (IC_50_ = 16.9 µM) exhibited reduced levels of these xenobiotics (Fig. 2).

### Retention of ML-EtBDP in *L. tropica* strains

**Fig 3:**
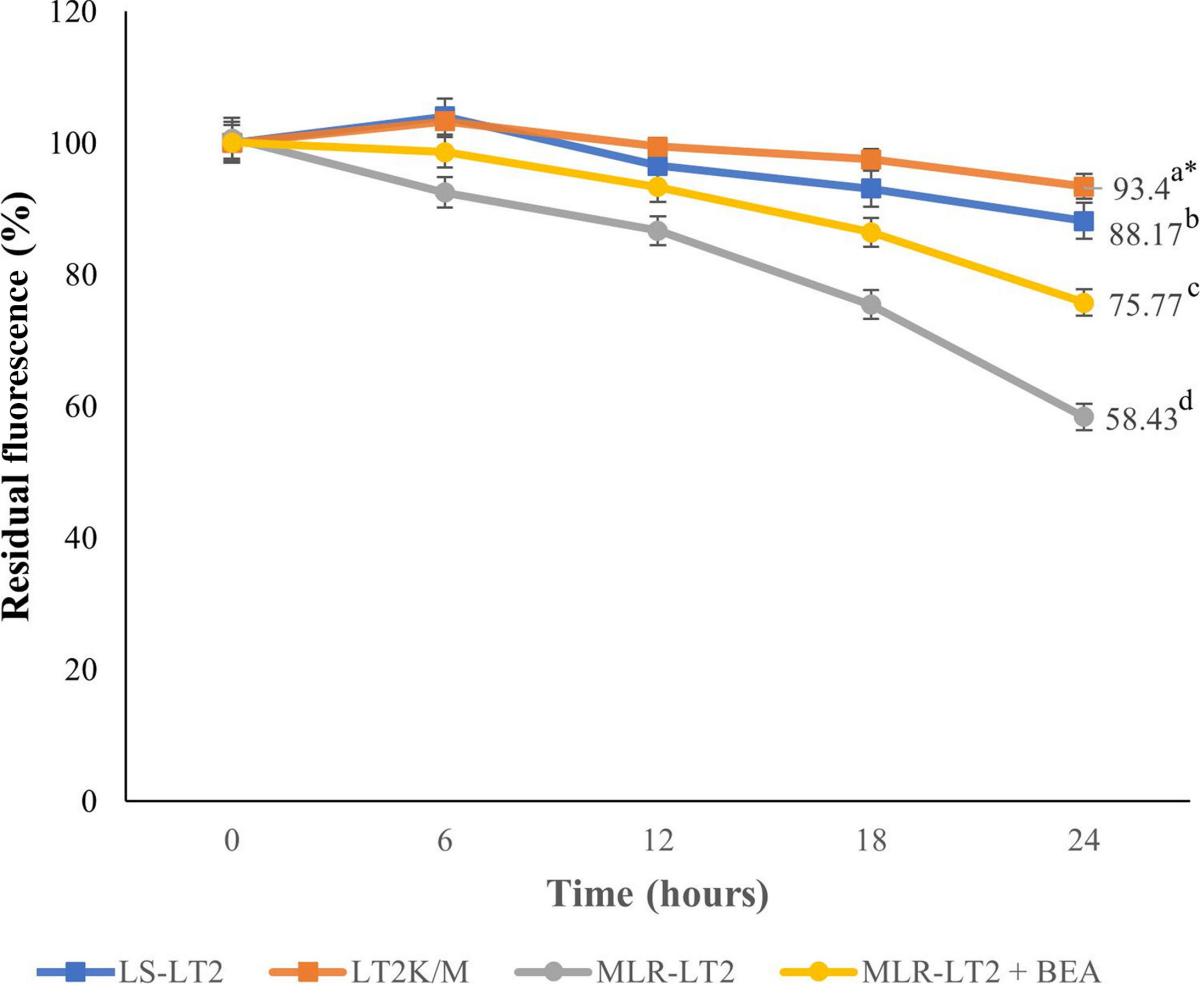
ML-EtBDP residual fluorescence in *L. tropica* strains LS-LT2, LT2^K/M^, and MLR-LT2 in the presence and absence of beauvericin (10 nM). Following the assessment of initial uptake, the fluorescence was investigated at 0, 6, 12, 18, and 24 h. The fluorescence intensity at each time point was standardized based on the initial uptake of ML-EtBDP, as variations in drug uptake were observed among these strains. One-way analysis of variance was utilized to evaluate the statistical significance of variations in residual fluorescence levels among distinct *Leishmania* strains in the presence or absence of beauvericin. *The values bearing different superscript letters within a column exhibit significant differences (*P* < 0.05).

The investigation into the prolonged intracellular presence of ML involved the quantification of ML-EtBDP fluorescence. We conducted a time-resolved analysis over intervals of 6, 12, 18, and 24 h for LS-LT2, LT2^K/M^, and MLR-LT2 strains (Fig. 3). As the experiment progressed, a noticeable pattern in ML retention became evident (df = 3, *F* = 334, *P* < 0.05). Remarkably, after 24 h, the MLR-LT2 strain exhibited a significant reduction to 58.4% in ML-EtBDP fluorescence compared to the initial uptake at 0 h. In contrast, LS-LT2 showed a more modest decrease to 88.16% compared to the initial uptake after the same duration (Fig. 3). Expectedly, after impairing the function of ABCG6, the LT2^K/M^ strain showed a negligible reduction of 93.43%. To assess the impact of BEA on the intracellular retention of ML, we quantified the ML-EtBDP fluorescence in the MLR-LT2 strain exposed to sub-lethal concentrations of BEA. Interestingly, the fluorescence exhibited a higher retention compared to the same strain treated with ML-EtBDP alone. These results demonstrate that the presence of BEA within the intracellular environment significantly extended the retention of ML.

### ATPase activities in plasma membranes

**Fig 4:**
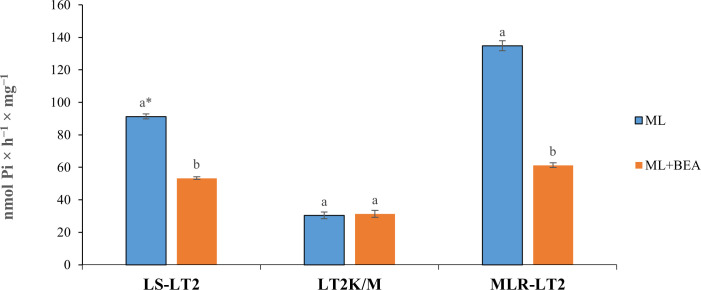
Effect of ML on ATPase activity of LS-LT2, LT2^k/M^, and MLR-LT2 strains of *L. tropica* in the presence or absence of beauvericin. Data are the means ± standard error of three replicates. One-way analysis of variance was utilized to evaluate the statistical significance of variations in the concentration of pi among distinct *Leishmania* strains in the presence or absence of beauvericin. *The values bearing different superscript letters within a column exhibit significant differences (*P* < 0.05).

In our studies of ATPase activities in *L. tropica*, we utilized rescued amastigotes from the LS-LT2, LT2^K/M^, and MLR-LT2 strains (overexpressing ABC transporter genes, predominantly *ABCG6*) (Fig. 4). To assess the neutralizing capability of BEA, we measured ATPase activity in the presence of the substrate ML. In LS-LT2, the baseline ATPase activity was determined to be 91.3-nmol Pi/h/mg. Remarkably, this activity decreased significantly to 53.3-nmol Pi/h/mg upon the introduction of BEA (df = 1; *F* = 4,228; *P* < 0.05). Strikingly, no significant difference was detected before (30.3 Pi/h/mg) and after the introduction of BEA (31.36 Pi/h/mg) in LT2^M/K^ (df = 1, *F* = 1.152, *P* > 0.05) (Fig. 4). Subsequently, in experiments involving the overexpression of ABC transporter genes (MLR-LT2), a trend similar to LS-LT2 was observed. The ATPase activity, initially at 134.8-nmol Pi/h/mg was significantly reduced to 61.26-nmol Pi/h/mg after the introduction of BEA (df = 1; *F* = 1,354; *P* < 0.05) (Fig. 4).

### Homology modeling and molecular docking

**Fig 5:**
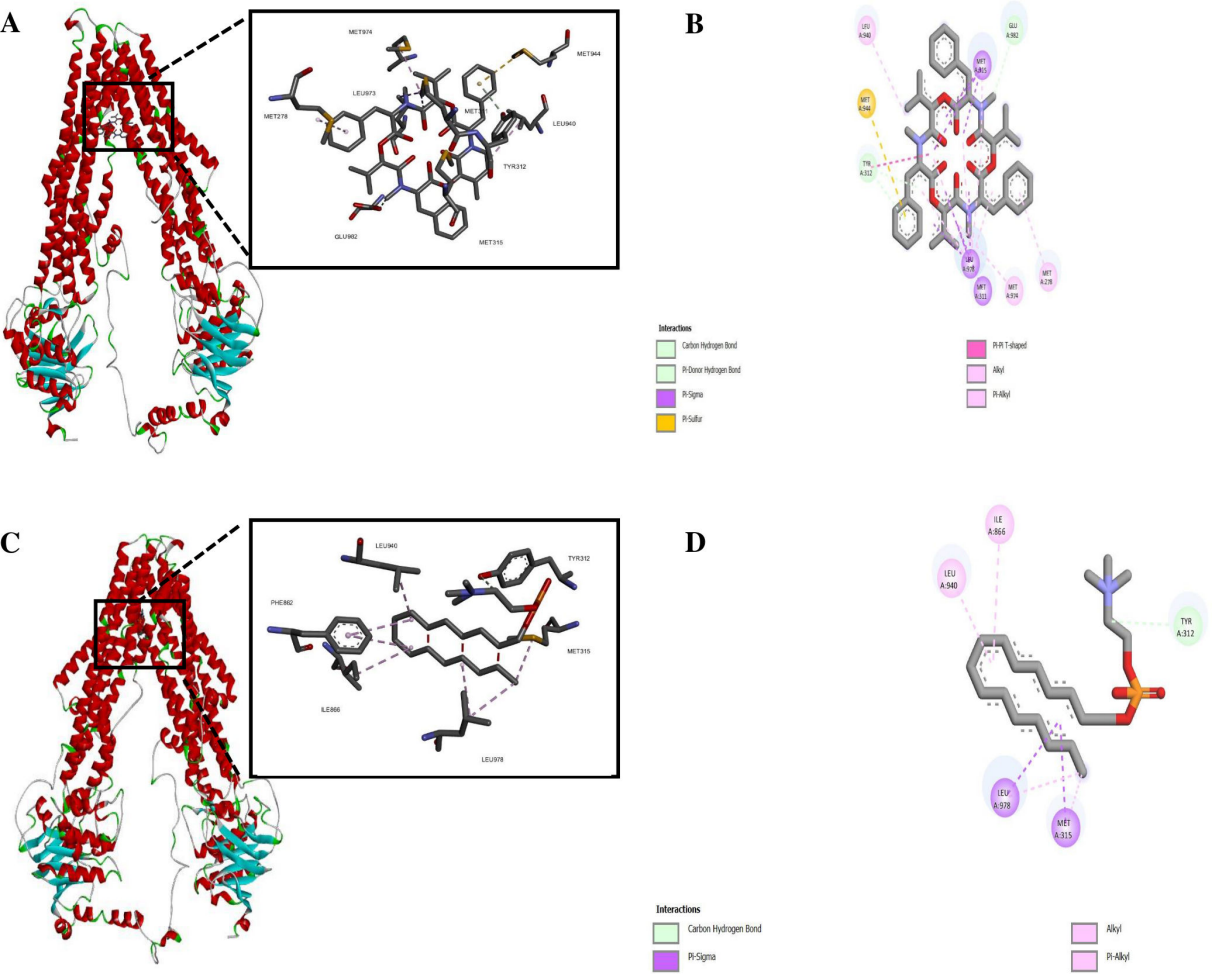
Molecular docking of BEA with ABC transporter: (**A**) surface representation of protein in red and BEA is shown in yellow and (**B**) two-dimensional (2D) diagram of interacting residues. Molecular docking of ML with ABC transporter. (**C**) Surface representation of protein in red and ML in yellow and (D) 2D diagram of interacting residues.

We utilized template-based modeling to predict the structure of the ABC transporter ABCG6. The quality of the model was evaluated at both the global and local levels, and it demonstrated a favorable MolProbity score. Structure validation was confirmed through the generation of a Ramachandran plot (Table S2). Intriguingly, BEA showed the lowest binding affinity with the ABC transporter relative to the ML (Table 3) while having a better docking ability compared to the controls QZ59RRR, QZ59SSS, and verapamil (Table 3). Hydrophobic interactions were formed between BEA and Met278, Met311, Tyr312, Leu940, Met974, and Leu978. In addition, BEA established H and S bonds with Glu982 and Met315, respectively (Fig. 5). In parallel, ML was linked to the protein through four hydrophobic interactions (Met315, Ile866, Leu940, and Leu978) and one H bond (Tyr312) (Fig. 5). BEA was associated with residues in TM5 (Met278), TM6 (Met311, Tyr312, and Met315), TM11 (Leu940), and TM12 (Met974, Leu978, and Glu982). ML solely interfaced with TM6 (Tyr312 and Met315), TM10 (Ile866), and TM12 (Leu940 and Leu978).

**TABLE 3:** ICM scores for ML, BEA, and different known inhibitors against the DBS of *L. tropica* ABC transporters (ABCG6)

Protein	Drug^*a*^	ICM score (kcal/mol)
ABCG6	ML	−11.17
	BEA	−15.62
	Verapamil	−12.43
	QZ59RRR	−12.87
	QZ59SSS	−14.8

^*a*^ BEA, beauvericin; ML, miltefosine.

### Dynamic simulation

**Fig 6:**
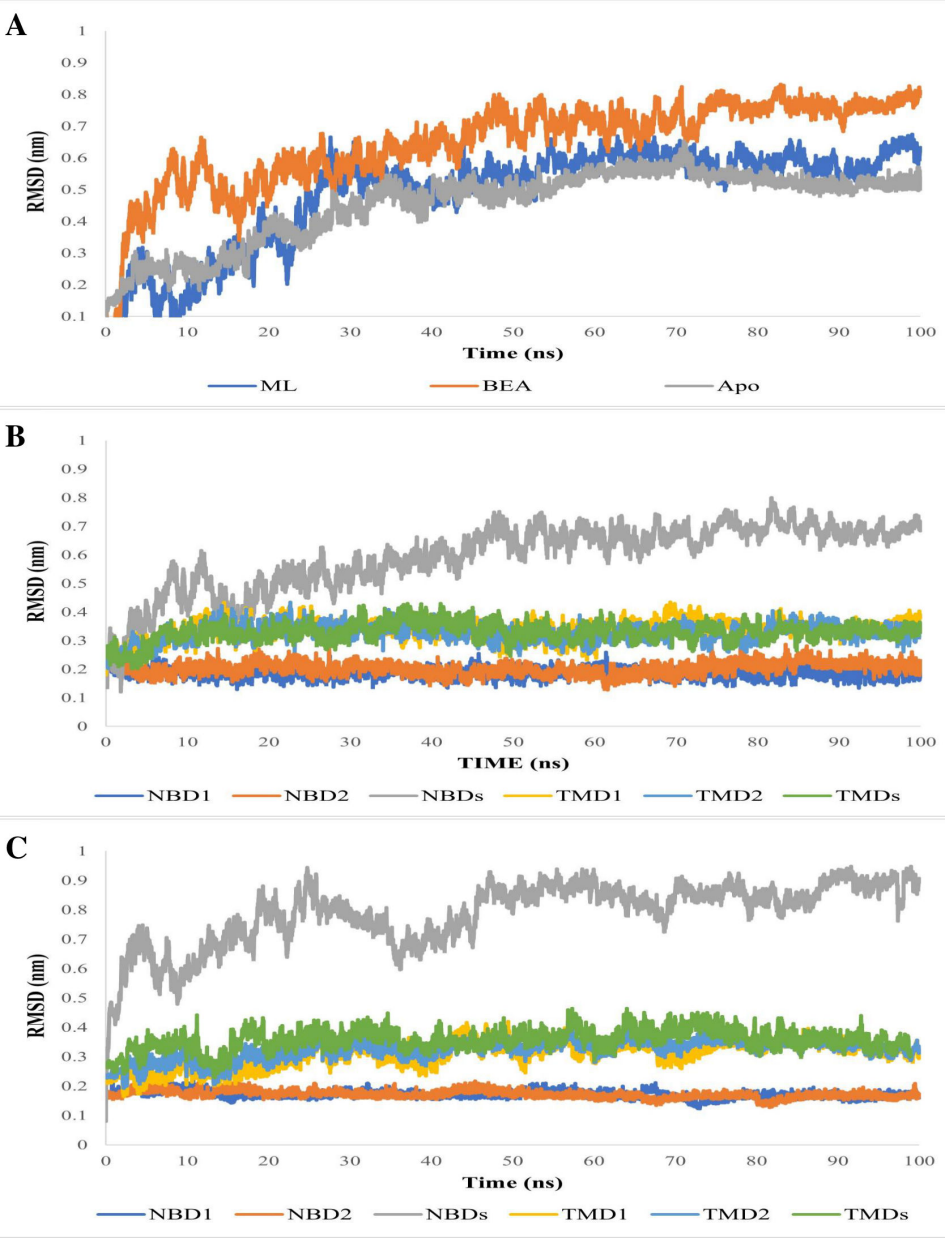
(**A**) Plots of the Cα RMSDs of ABC transporter (apo and in complex with ML and BEA) overall structures during 100 ns of MD simulation period. (**B**) Plots of the Cα RMSDs of ABC transporter individual domains in complex with ML. (**C**) Plots of the Cα RMSDs of ABC transporter individual domains in complex with BEA.

**Fig 7:**
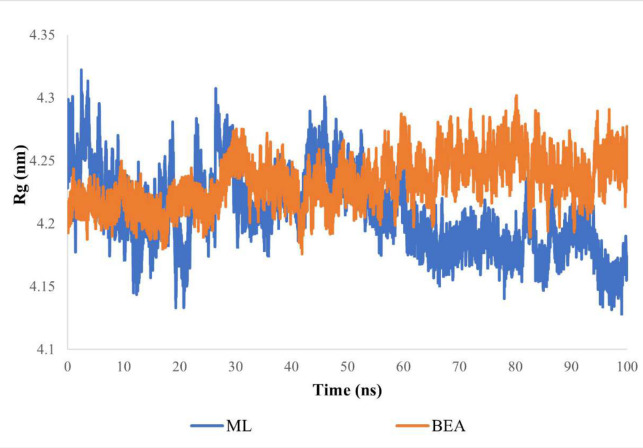
Radius of gyration of ABC transporter in complex with ML and BEA during 100 ns of the MD simulation period.

To further understand the mechanism through which BEA impedes the activity of efflux pumps, we simulated for 100 ns the conformational dynamics of a typical ABC transporter in a membrane environment (apo, substrate, and inhibitor bound). The tested systems displayed a rapid jump in the first 20 ns of the simulation, but overall, stable fluctuation over the remaining course of the trajectory was seen (Fig. 6), and all systems reached equilibrium, confirming that the simulation time was sufficient. The RMSD values for the free and substrate- and inhibitor-bound protein were 0.45, 0.48, and 0.64 nm, respectively (Fig. 6). Subsequently, we calculated RMSDs for TMDs and NBDs separately (Fig. 6). The TMDs exhibited a relatively stable RMSD of 0.3 nm, while the NBDs showed RMSD values ranging from 0.5 to 0.8 nm across all simulations. Cα RMSDs of individual domains were calculated separately for both complexes (MBA and BBA). Curiously, elevated and reduced fluctuations were notable in NBDs and TMDs, respectively (Fig. 5). Additionally, the Cα RMSD calculations indicated that NBD monomers (NBD1 and NBD2) were the most stable structures (Fig. 6). Additionally, we observed a consistently low Rg value of 4.23 nm for BBA over time, indicating no significant compression. In contrast, MBA exhibited a more compact conformation with an Rg value of 4.2 nm (Fig. 7).

### Binding free energy

The MM/PBSA is a reliable approach for estimating the small ligand-binding affinities to biological macromolecules. The inhibitor, BEA, showed lower (more favorable) free energies of binding (−149.5 kJ/mol) when compared to the substrate (−107.2 kJ/mol), ML (Table S4). The dominant contributors to the low binding energy in the inhibitor-bound simulation were van der Waals forces (Table S4). Additionally, all other binding free energy contributors were roughly comparable between BEA and ML-bound simulations (Table S4).

### Analysis of the ATP-binding domain

**Fig 8:**
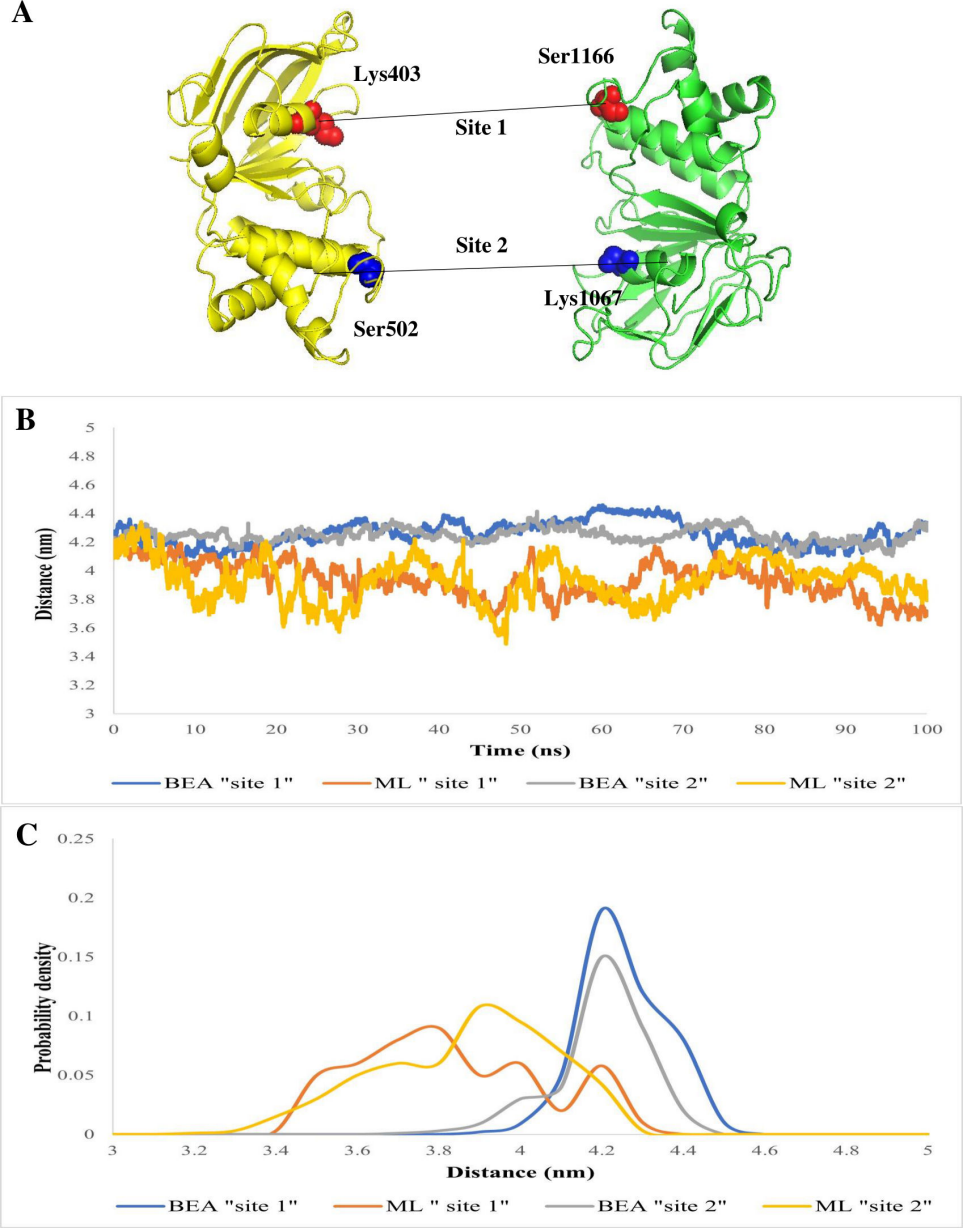
(**A**) Cartoon representation of ABC transporter nucleotide-binding domain displaying the conserved residue pairs at the nucleotide-binding site. The Cα-Cα distance of “site 1” formed between the N-terminal walker A motif (Lys403) and the C-terminal signature sequence (Ser1166) is shown in red, while that of “site 2” formed between the C-terminal walker A motif (Lys1067) and the N-terminal signature sequence (Ser502) is shown in blue. (**B**) Time series of the distances between the conserved residues in sites 1 and 2. (**C**) Distance distributions of sites 1 and 2 in the ABC transporter in complex with ML and BEA. The distributions were calculated every 0.5 Å.

To assess the integrity of NBS following substrate and inhibitor binding, we monitored Cα-Cα distances between the conserved residues located at separate halves of the NBD (Fig. 8). These distances in sites 1 and 2 showed moderate changes, with decreases observed after ML attachment and increases following BEA attachment (Fig. 8). In the ligand-bound state, the distance distribution in sites 1 and 2 exhibited heterogeneity for the substrate and homogeneity for the inhibitor (Fig. 8). Thereafter, we characterized the impact of these NBD conformational changes on NBS by calculating the differences in Cα atoms, backbone atoms, and heavy atoms between the four reference structures and MBA/BBA. In the case of MBA (RMSD 1.2 Å), both NBSs adopted a conformation more similar to the NBS of the four reference ABC transporters than to BBA (RMSD 1.5 Å). Molecular docking results revealed relatively favorable binding energy for ATP to the MBA (−5.2 kcal/mol) compared to BBA (−2.3 kcal/mol).

## DISCUSSION

Leishmaniases are a group of different diseases caused by the protozoan parasites belonging to the *Leishmania* spp., affecting a wide range of mammals, including humans (59). While infections can occur across various socioeconomic groups, they are more prevalent in areas lacking proper sanitation (7). Traditional control strategies involve several local and systemic treatments. However, the currently available medications suffer from limitations such as cost, availability, and effectiveness in disease management, exacerbated by the emergence and spread of drug resistance. The reduced susceptibility of *Leishmania* to drugs further complicates treatment and places a significant burden on healthcare systems (11, 60). In the course of our investigation, we systematically observed that the deliberate selection of a drug-resistant *L. tropica* strain resulted in a progressive increment in the IC_50_ values for ML against promastigotes. These values steadily escalated from 1.5 to 30.83 µM over a span of 15 rounds of selection. Parallelly, the IC_50_ of ML against axenic amastigotes underwent a similar augmentation, elevating from 1.1 to 48.17 µM during the same number of rounds of selection. Intriguingly, axenic amastigotes exhibited a heightened susceptibility to ML when compared to their promastigote counterparts, consistent with earlier observations reported by Zghair (61). Conversely, our examination unveiled that the intracellular amastigote form displayed a diminished susceptibility to ML treatment, characterized by lower susceptibility indices and RRs in contrast to the extracellular forms. One of the fundamental determinants of our limited understanding of drug resistance in *Leishmania* is that most studies have only focused on the promastigote, the clinically irrelevant form of the parasites (20). Despite its shortcomings, the experimental induction of drug resistance in promastigotes is considered less challenging when compared to the intracellular forms. Nonetheless, it is crucial to mention that resistance data obtained from the flagellated stage of the parasite should be processed with caution as it is significantly different compared to the intracellular amastigotes in terms of biology and drug susceptibility. To address these limitations, we previously developed an assay for the absolute quantification of gene expression using RT-qPCR to assess the therapeutic index of ML against all developmental stages of *L. tropica* (26). In addition, the resistance selection assay we used in this study with intracellular amastigotes was closely linked to the one developed by Hendrickx et al. (62). The back transformation of intracellular amastigotes into promastigotes was performed by replacing the RPMI medium with a MEM (minimum essential medium)-based medium (62). In our straightforward procedure, we relied, specifically, on SDS for the controlled lysis of amastigote-infected macrophage. All things considered, future studies should follow in our footsteps and assess the resistance development of *Leishmania* spp. in the extracellular as well as the intracellular forms.

ML stands as the sole approved oral treatment for leishmaniasis, yet our comprehensive understanding of its precise mechanism remains partially elucidated (63). However, it is firmly established that ML integrates itself into the parasite’s plasma membrane, consequently modifying membrane permeability and inducing perturbations in lipid metabolism (64). These intricate actions collectively culminate in programed cell death (64). It is noteworthy to emphasize that intracellular drug accumulation constitutes a pivotal prerequisite for the activation of ML’s mechanism of action (64). In this study, labeled ML and phosphocholine were quantified in strains of *L. tropica*, encompassing both susceptible and resistant variants, employing flow cytometry. The outcomes divulged dissimilar accumulation patterns for ML-EtBDP and PC-BODIPY between LS-LT2 and MLR-LT2. Intriguingly, this divergent accumulation displayed an inverse correlation with the IC_50_ values corresponding to each strain. These results are consistent with previous research conducted by Espada et al. (16). Their investigation revealed significant reductions in the uptake of ML (and phosphocholine) in ML-tolerant strains (LTCP 16907 and LTCP 19446) of *Leishmania braziliensis* when compared to the ML-sensitive strain (LTCP 16012). The association between the uptake of ML and the susceptibility of the parasite has been observed in numerous previous investigations involving *Leishmania donovani* (65), *Leishmania infantum* (66–68), and *Leishmania amazonensis* (69). The uptake of ML is facilitated by a translocation machinery that includes a P-type ATPase known as the *Leishmania* MLT. MLT’s functionality is closely associated with its interaction with Ros3, a specific B sub-unit of MLT belonging to the CDC50/LEM3 protein family (63). It could be postulated that the functionality of this intracellular transport system is compromised or disrupted in the resistant strains of *Leishmania*. Interestingly, even isolates with an intact ML transport pathway displayed a reduced intracellular accumulation (65, 70). The molecular underpinnings behind this diminished ML responsiveness were elucidated through an investigation into the dynamic expression patterns of *L. tropica*’s translocation machinery. Within MLR-LT2, the *Ros3* gene exhibited a substantial reduction in the abundance of *Ros3*-mRNA. Interestingly, this decline in expression was not mirrored in the transcripts of *MLT*, despite the apparent interdependence of the components constituting the MLT-Ros3 complex. Previous investigations have elucidated that Ros3 functions as a limiting factor in the transport rate of MLT-Ros3 in *L. braziliensis* (71). Intriguingly, the overexpression of the *Ros3* gene resulted in elevated levels of both MLT and Ros3 within the parasite’s membrane. These insights provide a plausible explanation for the lack of significant downregulation in *MLT* transcripts observed in resistant strains, a phenomenon similarly documented (16). In accordance with prior investigations (72–74), it is evident that the fitness levels of *Leishmania* strains exhibiting resistance to ML were notably diminished in comparison to their laboratory-susceptible counterparts. It has been previously established that the disruption of the MLT-Ros3 complex can exert influences on phospholipid uptake and cytoplasmic membrane structure, thereby imparting consequential effects on parasite fitness. The precise factors contributing to ML treatment failure remain a subject of ongoing investigation. Emerging evidence underscores a multifaceted interplay of factors, placing substantial emphasis not only on reduced drug accumulation (16, 63, 64) but also on the bolstered drug efflux mechanisms (15, 64, 75, 76). The investigation into fluorescence decay in ML-treated parasites demonstrated notable variations between LS-LT2 and MLR-LT2 (Fig. 3), indicating differences in efflux mechanisms among these parasite populations. It may be hypothesized that the heightened efflux observed in the artificially selected strain could be partly accountable for the reduced susceptibility to ML. Remarkably, the ML-treated LT2^K/M^ strain overexpressing the non-functional ABC transporter experienced a poor efflux capability compared to LS-LT2. This provided the first physical evidence that ABC transporters in general, specifically ABCG6, play an important role in the efflux of ML in *L. tropica*.

Moreover, we showed in this study that BEA could reverse resistance development and partially restore ML efficacy against *Leishmania* (Fig. 3). The ability of BEA to restore the effectiveness of therapeutic agents was previously demonstrated. Szczepaniak et al. (77) showed that BEA can increase the uptake of all anti-fungal drugs in *C. albicans*. Moreover, Tong et al. (22) revealed that BEA resensitizes *C. albicans*, *Aspergillus fumigatus*, and *Cryptococcus neoformans* against numerous azoles and could reverse multidrug resistance in *T. urticae* (23). ABC transporters are pivotal in the process of drug efflux across various organisms, including *Leishmania* parasites (20). These ubiquitous transmembrane proteins serve as versatile molecular pumps with the capacity to transport a diverse array of molecules, including drugs, across cellular membranes (20). In this study, we analyzed the transcription dynamics of ABC transporters, which play a key role in reducing the bioavailability of drugs. A previous *in silico* genome survey of *Leishmania* parasites revealed the presence of all eight known ABC transporter sub-families (A–H) (20). The sub-families ABCD, ABCE, ABCF, and ABCH were not detected in *Leishmania*. ABCA4 and ABCA8 have been previously described, and no treatment failure has been attributed to these proteins (78, 79). Our findings demonstrate that after each passage, the transcription level of the ABC transporters increased, and the drug’s effectiveness decreased, with the members of the ABCG sub-family being among the most differentially expressed in all life stages after 15 rounds of selection stress (Fig. 1). These results are in accordance with a previous report by Castanys‐Muñoz et al. (80), which showed that the overexpression of the *ABCG4* gene in *L. infantum* was associated with resistance to sitamaquine and ML and their analogs, edelfosine and perifosine. These observations were further supported by Castanys‐Muñoz et al. (81), who demonstrated that overexpression of *ABCG6* reduced the responsiveness of *L. infantum* to treatment with ML, camptothecin, sitamaquine, and chloroquine. The ABCG sub-family, also known as the white family, comprises six members in *Leishmania*. Among these, ABCG4 and ABCG6 have been previously associated with drug resistance. These two members are strategically located in the plasma membrane and flagellar pocket of *Leishmania* spp. Intriguingly, studies by Castanys‐Muñoz et al. (81) demonstrated that parasite transfectants overexpressing ABCG4 exhibited a diminished intracellular presence of ML. This finding is in concordance with the work of BoseDasgupta et al. (82), who reported that the transfection and overexpression of ABCG6 in promastigotes and axenic amastigotes of *L. donovani* resulted in reduced sensitivity to ML. These collective observations underscore the significant roles played by ABCG4 and ABCG6 in modulating the intracellular dynamics of ML, highlighting their potential as key players in the development of drug resistance in *Leishmania*. The ABC transposome of *Leishmania* comprises four members of the ABCB sub-family, represented by two “half” (ABCB1 and ABCB3) and two “full” transporters” (ABCB2 and ABCB4) (20). ABCB1 and ABCB3 have been shown to play a major role in the survival of trypanosomes but are not closely linked to anti-microbial resistance (83, 84). ABCB2 and ABCB4, respectively known as MDR2 and MDR1, have been designated as efflux transporters for leishmanicidal molecules (85–87). In this study, MDR1 and MDR2 were stably expressed in all developmental stages of the parasite (Fig. 1), which contradicts previous findings that suggested their role in the development of resistance in *Leishmania* spp. against vinblastine, daunorubicin, puromycin, driamycin, and doxorubicin (88–92). It is noteworthy that in *Leishmania*, MDR1, and MDR2 are primarily found in sub-cellular regions such as the Golgi apparatus, endoplasmic reticulum, multivesicular tubule lysosome, and mitochondria (20). The exact mechanism of action of ML against *Leishmania* remains unclear. However, a growing body of evidence suggests that ML mainly targets the cell membrane, which is its primary site of activity (93, 94), and could be attributed to the fact that the ML site of action is not in proximity to ABCB2 and ABCB4 intracellular localization. On the other hand, Seifert et al. (95) showed that *ABCB4* was stably expressed in ML-resistant strains of *L. donovani*, while ML-resistant *L. brazilensis* did not carry the *ABCB4* gene (71). This correlates with our results and further supports the idea that the ABCB sub-family contributes minimally to ML resistance in *Leishmania* spp. Numerous ABCC members were described in *Leishmania*, but only *ABCC3* and *ABCC7* were directly linked to treatment failure (20). A limited number of studies investigated the role of the ABCC sub-family in MDR development. *ABCC3* was overexpressed in clinical isolates of *L. donovani* resistant to pentavalent antimonial (96, 97), while no changes were detected in *ABCC3* and *ABCC7* transcription levels (98, 99). We also detected a stable *ABCC3* transcription profile in the intracellular forms of the parasite (Fig. 1). Initially, we hypothesized that *ABCC3* did not have a role in the translocation of ML. However, we detected a significant upregulation in its expression after 15 rounds of selection of promastigotes and axenic amastigotes. A concentration-dependent substrate modulation of ABC transporters was detected for the first time in this study, and the regulation of the ABC transporter genes was developmentally stage dependent. Remarkably, the *ABCC7* gene was overexpressed in the intracellular forms of *L. tropica* while stable in the extracellular stages (Fig. 1). The ABCC proteins play a pivotal role in transporting harmful substances into intracellular compartments, contributing significantly to cellular detoxification (100). This sub-family is known for sequestering toxic molecules within cells and aiding in their secretion, thereby playing a crucial role in cellular detoxification across diverse eukaryotic cells (100). The multifaceted functions of ABCC proteins underscore their importance in maintaining cellular homeostasis and defending against potentially harmful compounds. Particularly noteworthy within this sub-family is the sub-cellular localization of ABCC3, predominantly found in vesicles positioned between the nucleus and the flagellar pocket, while ABCC7 is situated in intracellular vesicles. These distinct sub-cellular distributions emphasize the varied and specialized functions of ABCC proteins in cellular processes. In line with this, there is a notable overexpression of ABCC3 in *L. infantum* axenic amastigotes exhibiting resistance to antimony (100). Importantly, our study contributes an important revelation, marking the first association of the ABCC sub-family with ML resistance. Additionally, it is crucial to note that our investigation represents the first comprehensive assessment of ABC transporter expression across the three developmental stages of *L. tropica*, considering exposure to ML and the implementation of 15 rounds of selection to generate the resistant clone. Understanding the role of ABC transporters in *Leishmania* is crucial for identifying mechanisms of drug resistance, given the intrinsic differences noted in resistance development. This variability arises from the interplay between *Leishmania* spp., developmental stages, and the drug’s pharmacokinetics and pharmacodynamics. Further investigations should extend beyond transcript studies to explore the corresponding proteins, particularly in an organism with intricate post-translational modifications.

In light of the aforementioned investigations that elucidated the dynamic transcriptional landscape associated with the acquisition of resistance in *L. tropica* to ML, it becomes evident that ABC transporters assume a pivotal role in this intricate process, marked by substantial alterations in their expression patterns. Recognizing ABC transporters as established targets of BEA, we undertook an investigative approach to evaluate ATPase activity in LS-LT2, LT2^K/M^, and MLR-LT2 strains, in the presence and absence of BEA. The first notable observation is that in the MLT-LT2 strain, the ATPase activity in the absence of BEA was significantly higher compared to the LS-LT2 counterpart. Here, the observed elevation in the parasite’s ATPase activity following exposure to excessive ML doses sheds additional light on the involvement of ABC transporters in the efflux of ML (Fig. 4). The second remarkable finding underscores the impact of BEA on ABC transporters, particularly evident in the LS-LT2 and MLR-LT2 strains, where a significant decrease in ATPase activity followed the introduction of BEA. This reduction aligns with the notion that BEA effectively targets functional ABC transporters, hampering their ATPase activity, a crucial element in their efflux function. Moreover, the results obtained from the LT2^K/M^ strain, overexpressing a non-functional ABCG6, revealed no significant difference in ATPase activity in the presence or absence of BEA. This observation further supports the specificity of BEA in targeting functional ABC transporters. In strains where the ABC transporter is rendered non-functional, as in the case of LT2^K/M^, the expected impact on ATPase activity is notably absent, indicating the selectivity of BEA in inhibiting the ATP hydrolysis, an essential step facilitating the energy-dependent conformational changes (from inward to outward facing) and the active transport process, ultimately leading to drug efflux from the cell (Fig. 4).

It is noteworthy that BEA application did not fully restore ML susceptibility in *L. tropica*, as other enzymes such as the cytochrome P450 (CYP450) could also be involved in mediating resistance. CYP450 enzymes, classified as hemoproteins, serve as essential catalysts in orchestrating a wide array of chemical processes. Their enzymatic functions encompass drug transformation, the alteration of foreign substances, the metabolism of potentially harmful chemicals, and the synthesis of vital biological molecules (101). In our investigation, we have showcased an increased expression of the *CYP450* gene across all life stages of the parasite. This observation implies a potential role for CYP450s in the metabolic pathways of ML. These outcomes are consistent with the findings of Verma et al. (102), who reported that the knockout of *CYP5122A1*, a novel CYP450 in *L. donovani*, results in increased susceptibility to pentavalent antimony and ML (102). Furthermore, the observed overexpression of *CYP5122A1* suggests its involvement in drug resistance mechanisms (102). Within the intricate machinery of cells, a class of proteins, known as heat shock proteins (Hsps), plays a multifaceted role. These versatile biomolecules are essential for ensuring the proper folding and functioning of other proteins (103). Most intriguingly, these proteins possess the capability to influence significant events within apoptotic pathways, thereby intervening in the initiation of programmed cell death (103). In this study, we have shown that an upregulation of Hsp (*HSP83*, *HSP60*, and *HSP70*) expression occurs in the resistant strain (MLR-LT2), potentially contributing to the parasite’s ability to evade the effects of ML. This aligns with prior findings, which have also indicated that a substantial portion of differentially expressed proteins in functional categories within *L. infantum* are associated with chaperones and stress-related proteins (104). In the context of *Leishmania*, S-adenosylmethionine synthetase (SAMS) plays a pivotal role in catalyzing the synthesis of S-adenosylmethionine (SAM), an indispensable molecule involved in a spectrum of vital biological processes, including gene expression, energy metabolism, and cell proliferation (105). Interestingly, previous research has consistently reported the upregulation of enzymes participating in the trypanothione biosynthesis pathway in *Leishmania* strains exhibiting resistance to antimonials and methotrexate (105, 106). However, our investigations have unveiled a reduction in the transcript levels of *SAMS* within the promastigote stage of MLR-LT2. Intriguingly, this reduction was not as pronounced in intracellular amastigotes. These findings align with the research conducted by Carnielli et al. (104), who similarly observed the downregulation of SAMS in ML-resistant isolates of *L. infantum*. It may be hypothesized that, while the downregulation of SAMS initially hints at a vulnerability due to reduced SAM availability, the intricate interplay of *Leishmania*’s antioxidant defense systems may trigger a compensatory response, bolstering protection against reactive oxygen species. This compensatory mechanism could contribute significantly to the survival of ML-resistant *Leishmania* strains. Furthermore, we noted a significant elevation in the transcript levels of proliferative cell nuclear antigen (PCNA) in MLR-LT2. PCNA plays a crucial role in DNA replication and repair processes. These results are consistent with prior research that has documented *PCNA* upregulation in clinical isolates of *L. donovani* resistant to antimonials (107) and *L. braziliensis* strains made resistant through antimonial exposure (108). These findings suggest a plausible link between increased *PCNA* expression and the emergence of drug resistance in *Leishmania* parasites. In addition, cytosolic peroxiredoxins (PRX1A) play a crucial role in antioxidant defense and redox regulation in *Leishmania* (108, 109). Concurrently, the heightened presence of reactive oxygen species induced by ML exposure, resulting in oxidative stress and subsequent cell death, appears to be offset by the elevated abundance of *PRX1A*.

Molecular docking presented a first line of evidence on the potential role of BEA against protozoan ABC transporters. The observed substantial upregulation of *ABCG6* following exposure to ML indicates its heightened involvement in the transport process and suggests a potential key role in mediating ML resistance. Focusing on ABCG6 for detailed molecular investigations, we aim to unravel the mechanisms behind its substrate and inhibitor interaction, providing valuable insights for understanding and potentially targeting the drug transport process in *L. tropica*. It is noteworthy that despite the relatively higher ICM score (lower affinity), verapamil was shown to be effective in reversing resistance to arsenite (82), sodium stibogluconate (110), pirarubicin (111), and pentamidine (28, 112) in *Leishmania.* The most prominent observation emerging from the obtained data comparison was that the ABC transporter (ABCG6) established unique residue interactions based on the type of the attached ligand. Specifically, BEA exhibited interactions with residues in TM5, TM6, TM11, and TM12. In contrast, ML interacted solely with TM6, TM10, and TM12. These findings were consistent with previous results by Aller et al. (50), which showed that the binding cavity of P-glycoprotein could accommodate at least two compounds simultaneously. Ma and Biggin (113), on the other hand, reported significantly different internal dynamics of the protein based on the ligand docking position. Compared to the conserved NBD, the TMD of all ABC transporters contains several highly variable motifs (114), and as a result, *L. tropica* ABC transporter residues involved in substrate/inhibitor translocation are not well resolved. However, based on the results obtained in this study, it can be reasonably assumed that BEA impedes drug resistance by binding to critical residues involved in drug binding or the conformational transition from the inward- to the outward-facing orientation of the protein.

The simulation of the apo ABC transporter showed similar Cα RMSD values to previous MD studies over a 100-ns timescale (115–118). This suggests that even the apo (ligand-free) ABC transporter can undergo substantial movements. After confirming the validity of the simulation system employed in this study, we next studied the impact of the substrate (ML) versus inhibitor (BEA) on the trajectory. Overall, our findings suggested that the efflux pump exhibited large rigid-body motions between the two NBD monomers, which was in line with previous studies demonstrating that the NBDs move toward each other (113, 119). Interestingly, this large conformational change was detected in both the MBA and BBA simulations in the absence of ATP. The first set of analyses, comparing the motion patterns of the efflux pump after BEA and ML binding, involved monitoring the distance between conserved motifs of the monomeric NBDs. The binding motif distances were considerably smaller in the MBA (Fig. 7), suggesting that the two NBDs move toward each other, alternating from the open, substrate binding to the closed, substrate-effluxing conformation (120). Additionally, the attachment of ML to the DBS could induce conformational changes in the NBD from a disassociated unaligned open dimer to a closed symmetrical dimer. A similar pattern was also observed by Xing et al. (119), who indicated the presence of an intermediate conformation that can accelerate the dimerization process. In contrast, the binding motif distances were equivalent to those in the initial structure in the BBA simulation, suggesting that the efflux pump lingered in the inward-facing conformation. Besides, we have verified and reached a similar conclusion through assessing the compactness (Rg) of the protein-ligand complexes. Notably, the BBA showed no compression and fluctuated stably around an Rg of 4.23 nm, which elucidates that the inhibitor operated by holding the NBDs apart and ergo preventing ATP hydrolysis (Fig. 6). The Rg value of the ML-bound ABC transporter declined significantly in comparison with the initial structure, indicating a compression in the transmembrane protein after the attachment of the substrate. Alongside molecular docking, the MM/PBSA is a useful process that offers a more precise calculation of the drug-protein binding energy. According to our findings, BEA has the highest affinity for ABC transporters. On the one hand, van der Waals, electrostatic, and non-polar solvation contribute adversely to the overall binding energy, while polar solvation energy contributes favorably to the total interaction energy. Our findings suggested that hydrophobic interactions played a key role in stabilizing the BEA-ABC transporter complex. A higher number of hydrophobic interactions within the DBS are one of the primary characteristics that permit an inhibitor to competitively impede substrate binding or restrict conformational changes associated with the efflux. To provide a thorough insight into the impact NBD motions have on the NBS, we compared the crystal structures of the ATP-binding sub-unit of MBA and BBA to those of ABC transporters co-crystallized with ATP (Table S3). Based on structural similarity, the MBA showed a higher competency to bind ATP compared to the BBA. Further molecular docking tests concurred with our initial finding that the competency of NBS putative residues exhibits a ligand-dependent behavior. Of note, the MBA’s NBS adopts a geometry closer to the reference (co-crystallized) ABC transporter compared to that of the BBA. Considerable progress has already been made in understanding the efflux mechanism of ABC transporters, and an alternate cycle for ATP hydrolysis was suggested (47). After the drug binds to the DBS, it induces long-range allosteric changes in the NBD. Drug binding to the DBS, in conjunction with ATP binding to the NBS, can mediate the interconversion from the inward- to the outward-facing orientation. Consequently, the energy released by ATP hydrolysis is used to reset the conformation cycle. BEA could accordingly inhibit these interconversions by blocking ML binding, thereby interfering with efflux initiation. A major pitfall linked to several inhibitors is that they also can translocate through ABC transporters. As a result, the effective chemosensitizing dose of the inhibitor is usually too high, which could lead to adverse reactions (121). However, the effective BEA concentration was low compared to the substrate; it blocks the entrance of ML to the DBS and consequently the conformational changes needed for the initiation of the efflux process.

In recent times, the pharmaceutical industry has progressively directed its focus toward fungal secondary metabolites, primarily motivated by the broad spectrum of biological activities that these compounds manifest. This burgeoning interest stems from the recognition of fungal secondary metabolites as a potential source of novel pharmacological agents with diverse therapeutic applications (122). Nevertheless, it is imperative to underscore that, despite their promising utility, the safety profiles of these bioactive molecules remain significantly underexplored and inadequately characterized. The safety profile of BEA has undergone a comprehensive assessment investigating its cytotoxic potential across a spectrum of human cell lines (122). This extensive investigation yielded IC_50_ values as follows: HEK2 keratinocytes (5.4 µM), human intestinal cell line Caco 2 (3.9 μM), human liver cell line HEPG2 (3.4 µM), human normal vascular endothelial cells (2.4 µM) (122), and fibroblast skin cells (4.8 µM) (123). These findings serve as an essential foundation for our present inquiry, shedding light on the safety profile of BEA. Notably, our current investigation has unveiled a promising perspective, suggesting that the therapeutic index of BEA for combating cutaneous leishmaniasis infections may be exceptionally high. To further substantiate the safety profile of BEA, we have calculated its IC_50_ values against diverse leishmanial stages, encompassing promastigotes (0.24 µM), axenic amastigotes (0.25 µM), and intracellular amastigotes (0.34 µM), alongside macrophages (3.7 µM) (data not shown). These results affirm the notion that the effective concentrations required for the inhibition of ABC transporters are remarkably low in comparison to those determined for all tested human cell lines, thereby underscoring the potential safety of BEA in the context of leishmaniasis therapy. Rodríguez-Carrasco et al. (124) conducted additional assessments utilizing a robust and sensitive liquid chromatography tandem mass spectrometry methodology for the precise quantification and identification of BEA in both murine tissues and biological fluids. Collectively, these observations revealed an absence of toxicological manifestations throughout the mice’s lifespan, with no associated pathological alterations. Notably, BEA was well tolerated and detected in multiple tissues, including the liver, fat, colon, muscle, kidney, and brain, as well as serum and tumor tissues. These pharmacokinetic findings position BEA as a promising candidate for potential therapeutic applications in cancer treatment. Furthermore, there is a growing recognition of the potential of BEA for topical applications in the treatment of dermatological conditions such as eczema and psoriasis (125).

### Conclusion

This study represents a comprehensive investigation into the complex interactions and responses of *L. tropica* to ML resistance. We have, for the first time, elucidated the transcription profile of genes implicated in the acquisition of ML resistance within a laboratory-induced resistant strain of *L. tropica.* Crucially, we have demonstrated that BEA possesses the capacity to partially counteract ML resistance, rendering resistant *L. tropica* strains partially susceptible to treatment. Our approach extended to employing molecular docking and MD simulations to delve into the mechanistic aspects of this reversal. By scrutinizing the dynamic movement of atoms, we postulate that BEA may intervene in the conformational transition from an inward-facing to an outward-facing conformation. As a result, the application of combination therapy, involving both the anti-leishmanial drug and BEA, a potent ABC transporter inhibitor, exhibits the potential to enhance the therapeutic index of anti-leishmanial agents. This approach holds promise for impeding the development of drug resistance and ultimately improving clinical outcomes.

## SUPPLEMENTAL MATERIAL

Supplemental material aac.01368-23-s0001.docx Tables S1 to S9, Figures S1 to S7, and Fasta sequence of the amplicons generated by the primers designed in this study.

ASM does not own the copyrights to Supplemental Material that may be linked to, or accessed through, an article. The authors have granted ASM a non-exclusive, world-wide license to publish the Supplemental Material files. Please contact the corresponding author directly for reuse.
