# Supplementary material for: ABC transporter inhibition by beauvericin partially overcomes drug resistance in Leishmania tropica
Source: Antimicrob Agents Chemother. 2024 Apr 4;68(5):e01368-23. doi: 10.1128/aac.01368-23 (PMC11064568; doi:10.1128/aac.01368-23)

**Fasta sequence of the amplicons generated by the primers designed in this study**

**>*ABCB2***

GCCATGTCCGCCGATAAAACGGCGAAGGCCAAGCAAGCCATCCACGATGCGACGGCGGCAGTGTCCGAGAAGCTAAGGGGTCA

**>*ABCB4***

CCGTATCGCAACGGATCTCATGTCTGGCGTGGGCAGTGTCGAGCTCAGCGCGGCGAAGACGTCGTTGATCATGGTGTACGTCGGTATCGGCATG

>***ABCC3***

GGAAACGCTCAATTGGCTGGTGCGGCAGGTTGCGACGGTGGAGGCAAACATGAACAGCGTGGAGCGTGTGATGTATTACACCCACGAAGTGGAGC

>***ABCC7***

TTCCCTTTTTGGGCATTGCGGGATACGTCATGACGTCTTTAGCAGTGACGCTATACACGTCACCGCTTAGTGTGGTTGTGGTGCTGCTAGCGGCTTACGCTTTTGTGCGCCTGCTGAAGTTCTACGCGACAGTGGTG

>***ABCG4***

GGCGAGGAAGTAGGTGTACGCGTTGTAGGCGTCGTTCGCCTGCTCCTGCAGGAAGACGGCTCGCTCCGGCGGGAAGGCGGCAATACCGTTCATGGCAGCACCCAAAAGGTTGTT

>***ABCG6***

AAACATCTCAAGGCTGCCGAGGGAGGAGAAGGTACTGATCATCATGATGAAAAAGAGGACGCCCATGCGACTGCGGATGCTGTCCTGCGAGTTCCCGACTTGGTAGTAGATCGTGCCCGTG

> ***ML-T***

CAAGTACAGGGAAACGCCCTACCTCCGCTTCTTTATCAGCTTTCGCAAGAACGTAGCTCTGTGGGGGTACCGTTACTTGAGTTACTTCATTTTGTTGAGCTTCTGCGTGC

>***Ros3***

CCGTATCTCCGAGAGGGCTACTACTACAAAGAGCCTGGGCACAAGATCCCACTCAACACTGATGAGGATTTGATAGTGTGGCTCGATCCGG

> ***CYP5122A1***

TTCATGTGTCTCCTCGGCACCGGCCTGGTCACCTCAGAGGGTGAGCAGTGGAAGAAGGGGCGCCTGCTGTTGTCGCATGCGATGCGCATCGACATTCTGGACAGCGTCCCAGAAATGGCTA

> ***HSP83***

ATGAGCATGATCGGCCAGTTCGGTGTCGGCTTCTACTCGGCGTACCTTGTGGCGGACCGCGTGACGGTGACGTCGAAGAACAACTCCGACGAGTCGT

> ***HSP60***

AGGCCCGACAGCTGATACTATCTGGTATCGAGCGCATCGCCACTGCCGTCGGGGTAACACTCGGCCCCAAGGGCCGGAACGTGATAATCCGGCAACCGGACGGGGAGCCAAAGATCACCAA

>***HSP70***

AGGACGCGAAGATGGACAAGCGCTCCGTGCACGACGTGGTGCTGGTGGGCGGGTCAACGCGCATCCCGAAGGTGCAGTCCCTCGTGTCGGACTTCTTC

> ***PRX1A***

CTCTTCATCATCGACCCCCGTGGCATGGTGCGTCAGATCACCGTCAACGACATTCCGGTGGGCCGCAACGTGGAGGAGGCTCTGCGCCTGCTGGAGGCTTTGCAGTTCGTGGA

> ***SAMS***

GACATTGGCTTCGATTCCGCGGACAAGGGTCTGGACTACGAGTCGTGCAATGTGCTGGTTGCGATTGAGCAGCAGTCGCCGGACATCTGCCAGGGTCTGGGCAACTTCGATAGCGAGGA

> ***PCNA***

AGGAGCCCATCACACTCTCTTTTGCGCTCCGCTTCATGGGCATCTTTGCCAAGGGCTCGACGCTCAGCGAGCGCGTCACGCTCAAGTTTGCCAAGGACAGTCCTTGCATGGTGGAGT

**Table S1:** *Leishmania* primers used in this study

| **Gene** | **Protein**  **(Alias)** | **Sequence** | **Amplicon size** | **Amplification efficacy (%)** | **Reference** |
| --- | --- | --- | --- | --- | --- |
| *ABCB2* | ABCB2 (MDR2) | GCCATGTCCGCCGATAAAAC | 83 | 99.2 | This study |
|  |  | TGACCCCTTAGCTTCTCGGA |  |  |  |
| *ABCB4* | ABCB4 (MDR) | CCGTATCGCAACGGATCTCA | 94 | 100.5 | This study |
|  |  | CATGCCGATACCGACGTACA |  |  |  |
| *ABCC3* | ABCB3 (PGPA/MRPA) | GGAAACGCTCAATTGGCTGG | 95 | 100.3 | This study |
|  |  | GCTCCACTTCGTGGGTGTAA |  |  |  |
| *ABCC7* | ABCC7 (PRP1) | TTCCCTTTTTGGGCATTGCG | 137 | 101.1 | This study |
|  |  | CACCACTGTCGCGTAGAACT |  |  |  |
| *ABCG4* | ABCG4 | GGCGAGGAAGTAGGTGTACG | 114 | 99.8 | This study |
|  |  | AACAACCTTTTCGGTGCTGC |  |  |  |
| *ABCG6* | ABCG6 | AAACATCTCAAGGCTGCCGA | 121 | 102.3 | This study |
|  |  | CACGGGCACGATCTACTACC |  |  |  |
| *ML-T* | ML-T | CAAGTACAGGGAAACGCCCT | 110 | 100.7 | This study |
|  |  | GCACGCAGAAGCTCAACAAA |  |  |  |
| *Ros3* | Ros3 | CCGTATCTCCGAGAGGGCTA | 91 | 101.6 | This study |
|  |  | CCGGATCGAGCCACACTATC |  |  |  |
| *CYP5122A1* | CYP450 | TTCATGTGTCTCCTCGGCAC | 121 | 98.6 | This study |
|  |  | TAGCCATTTCTGGGACGCTG |  |  |  |
| *HSP83* | HSP83 | ATGAGCATGATCGGCCAGTT | 97 | 98.1 | This study |
|  |  | ACGACTCGTCGGAGTTGTTC |  |  |  |
| *HSP60* | HSP60 | AGGCCCGACAGCTGATACTA | 121 | 102.6 | This study |
|  |  | TTGGTGATCTTTGGCTCCCC |  |  |  |
| *HSP70* | HSP70 | AGGACGCGAAGATGGACAAG | 98 | 97.9 | This study |
|  |  | GAAGAAGTCCGACACGAGGG |  |  |  |
| *PRX1A* | PRX1A | CTCTTCATCATCGACCCCCG | 94 | 100.7 | This study |
|  |  | TCCACGAACTGCAAAGCCTC |  |  |  |
| *SAMS* | SAMS | GACATTGGCTTCGATTCCGC | 119 | 99.5 | This study |
|  |  | TCCTCGCTATCGAAGTTGCC |  |  |  |
| *PCNA* | PCNA | AGGAGCCCATCACACTCTCT | 117 | 98.8 | This study |
|  |  | ACTCCACCATGCAAGGACTG |  |  |  |
| kDNA |  | CCTATTTTACACCAACCCCCAGT | 116 | 98.04 | Yehia et al. 2012 |
|  |  | GGGTAGGGGCGTTCTGCGAAA |  |  |  |
| *GAPDH* | GAPHD | TCCCACCTTTCTCATCCAAG | 128 | 101.4 | Al Khoury et el. 2022 |
|  |  | CATCACCCCTCTACCTCCCT |  |  |  |
| *Actin* | Actin | CCTATTTTACACCAACCCCCAGT | 116 | 99.7 | Yehia et al. 2012 |
|  |  | GGGTAGGGGCGTTCTGCGAAA |  |  |  |

**Table S2:** Evaluation scores obtained for the target ABC transporter using Swiss-model and 3D-refine servers.

| **Gene ID** | **Template used for homology**  **modeling** | **Sequence identity (%)** | **Ramachandran score (% of**  **residues)** | **GMQE score** | **Mol probity** | **Ramachandran score after**  **refinement (% of residue)** |
| --- | --- | --- | --- | --- | --- | --- |
| *ABCG6* | 4F4C | 35.14 | 90.1 | 0.61 | 1.2 | 91.2 |

**Table S3.** Co-crystal structures of ABC transporters and ATP deposited in the PDB.

| **PDB ID** | **Resolution**  **(Å)** | **Description** |
| --- | --- | --- |
| 1L2T | 1.90 | Hypothetical ACB transporter MJ0796 |
| 1Q12 | 2.60 | Maltose/maltodextrin transporter malK |
| 2FGK | 2.70 | Alpha-hemolysin translocation hlyB |
| 2IXF | 2.00 | Antigen peptide transporter 1 |

**Table S4:** Average binding free energies calculated from the molecular dynamic (MD) simulation in triplicate.

| Complex | van der Waals (kJ/ mol) | Electrostatic (kJ/mol) | Polar energy (kJ/ mol) | Non-polar energy (kJ/mol) | Binding energy (kJ/mol) |
| --- | --- | --- | --- | --- | --- |
| BEA-ABC transporter | -165.4 ± 16.7 | -7.6 ± 0.7 | 38.9 ± 1.8 | -15.4 ± 1.1 | -149.5 ± 9.5 |
| ML-ABC transporter | -119.3 ± 11.3 | -6.5 ± 0.3 | 33.4 ± 1.7 | -14.8 ± 1.1 | -107.2 ± 7.7 |

**Dose response of intracellular amastigotes**

**Table S5**: Standards (10–10^9^) for kDNA from Ct values of standards.

|  | | | | | | | | | | | | | | | | | | | | | |
| --- | --- | --- | --- | --- | --- | --- | --- | --- | --- | --- | --- | --- | --- | --- | --- | --- | --- | --- | --- | --- | --- |
| Intracellular amastigotes (standard) | | | | | | | |  | |  | |  | |  | |  | |  | |  | |
| DNA copies/uL | Log DNA copies/uL | Ct (first repetition) | Ct (second repetition) | Ct (thrid repetition) | Average Ct | S.E. |  | |  | |  | |  | |  | |  | |  | |  |
| 10 | 1 | 34.33 | 34.5 | 34.65 | 34.49 | 0.16 |  | |  | |  | |  | |  | |  | |  | |  |
| 100 | 2 | 31.09 | 31.23 | 31.33 | 31.21 | 0.12 |  | |  | |  | |  | |  | |  | |  | |  |
| 1000 | 3 | 26.89 | 26.81 | 26.71 | 26.8 | 0.09 |  | |  | |  | |  | |  | |  | |  | |  |
| 10000 | 4 | 23.51 | 23.62 | 23.68 | 23.6 | 0.08 |  | |  | |  | |  | |  | |  | |  | |  |
| 100000 | 5 | 20.17 | 20.32 | 20.25 | 20.24 | 0.07 |  | |  | |  | |  | |  | |  | |  | |  |
| 1000000 | 6 | 17.09 | 17.28 | 17.19 | 17.18 | 0.09 |  | |  | |  | |  | |  | |  | |  | |  |
| 10000000 | 7 | 14.15 | 14.33 | 14.07 | 14.18 | 0.13 |  | |  | |  | |  | |  | |  | |  | |  |
| 100000000 | 8 | 10.58 | 10.69 | 10.73 | 10.66 | 0.07 |  | |  | |  | |  | |  | |  | |  | |  |
| 1000000000 | 9 | 7.28 | 7.38 | 7.15 | 7.27 | 0.11 |  | |  | |  | |  | |  | |  | |  | |  |

**Figure S1:** Standards (10–10^9^) plot for kDNA from Ct values of standards. Amplification

efficiency was 98.04% for kDNAbased on the following formula: E = 10^[-1/slope]^

**Table S6**: Dose-Response Profiling of Miltefosine on Intracellular Amastigotes of *Leishmania tropica* LS-LT2 (round of selection 1). This experiment was repeated three times in triplicate.

|  | Amastigotes (test samples - Replicate 1) | | | Amastigotes (test samples - Replicate 2) | | | Amastigotes (test samples - Replicate 3) | | |
| --- | --- | --- | --- | --- | --- | --- | --- | --- | --- |
| ML concentration (uM) | Ct | Log DNA (post-drug exposure) | Log DNA (dead cells)* | Ct | Log DNA (post-drug exposure) | Log DNA (dead cells) | Ct | Log DNA (post-drug exposure) | Log DNA (dead cells) |
| 0 | 10.7 | 7.94 | 0.00 | 10.72 | 7.93 | 0.00 | 11.69 | 7.65 | 0.00 |
| 0.1 | 11.06 | 7.83 | 0.11 | 11.08 | 7.83 | 0.11 | 11.04 | 7.84 | 0.10 |
| 0.5 | 11.97 | 7.56 | 0.38 | 11.94 | 7.57 | 0.37 | 11.46 | 7.72 | 0.23 |
| 1 | 12.82 | 7.31 | 0.63 | 12.76 | 7.33 | 0.61 | 12.50 | 7.41 | 0.53 |
| 1.5 | 13.86 | 7.00 | 0.94 | 13.9 | 6.99 | 0.95 | 13.81 | 7.02 | 0.92 |
| 2 | 15.87 | 6.41 | 1.53 | 15.85 | 6.41 | 1.53 | 15.60 | 6.49 | 1.45 |
| 2.5 | 18.61 | 5.59 | 2.35 | 18.67 | 5.58 | 2.37 | 18.35 | 5.67 | 2.27 |
| 3 | 21.66 | 4.69 | 3.25 | 21.65 | 4.69 | 3.25 | 21.64 | 4.69 | 3.25 |
| 3.5 | 27.59 | 2.93 | 5.01 | 27.63 | 2.92 | 5.02 | 27.18 | 3.05 | 4.89 |
| 4 | 35.12 | 0.69 | 7.25 | 35.11 | 0.70 | 7.24 | 35.31 | 0.64 | 7.30 |
| 4.5 | 35.91 | 0.46 | 7.48 | 36.02 | 0.43 | 7.51 | 35.42 | 0.60 | 7.34 |
| 5 | 36.82 | 0.19 | 7.75 | 36.88 | 0.17 | 7.77 | 35.81 | 0.49 | 7.45 |

*Log DNA (dead cells) was derived through the subtraction of the log DNA value (post-drug exposure) for cells exposed to a specific concentration of ML from the log DNA value (post-drug exposure) for cells not subjected to ML.

**Figure S2**: : Dose-Response Profiling of Miltefosine on Intracellular Amastigotes of *Leishmania tropica* LS-LT2 (round of selection 1). The curves were generated by plotting the log DNA (dead cells) in relation to the concentrations of the drug.

**Table S7**: Dose-Response Profiling of Miltefosine on Intracellular Amastigotes of *Leishmania tropica* LS-LT2 (round of selection 5). This experiment was repeated three times in triplicate.

|  | Amastigotes (test samples - Replicate 1) | | | Amastigotes (test samples - Replicate 2) | | | Amastigotes (test samples - Replicate 3) | | |
| --- | --- | --- | --- | --- | --- | --- | --- | --- | --- |
| ML concentration (uM) | Ct | Log DNA (post-drug exposure) | Log DNA (dead cells)* | Ct | Log DNA (post-drug exposure) | Log DNA (dead cells) | Ct | Log DNA (post-drug exposure) | Log DNA (dead cells) |
| 0 | 10.69 | 7.94 | 0.00 | 10.69 | 7.94 | 0.00 | 10.69 | 7.94 | 0.00 |
| 0.1 | 10.83 | 7.90 | 0.04 | 10.81 | 7.91 | 0.04 | 10.9 | 7.88 | 0.10 |
| 0.5 | 10.93 | 7.87 | 0.07 | 10.89 | 7.88 | 0.06 | 11.1 | 7.82 | 0.23 |
| 1 | 11.35 | 7.75 | 0.20 | 11.35 | 7.75 | 0.20 | 11.39 | 7.74 | 0.53 |
| 1.5 | 12.14 | 7.51 | 0.43 | 12.15 | 7.51 | 0.43 | 12.34 | 7.45 | 0.92 |
| 2 | 13.88 | 7.00 | 0.95 | 13.85 | 7.01 | 0.94 | 14.03 | 6.95 | 1.45 |
| 2.5 | 16.83 | 6.12 | 1.82 | 16.52 | 6.21 | 1.73 | 16.77 | 6.14 | 2.27 |
| 3 | 19.41 | 5.36 | 2.59 | 19.37 | 5.37 | 2.58 | 19.69 | 5.27 | 3.25 |
| 3.5 | 24.39 | 3.88 | 4.07 | 24.22 | 3.93 | 4.02 | 24.82 | 3.75 | 4.89 |
| 4 | 29.24 | 2.44 | 5.50 | 29.12 | 2.47 | 5.47 | 29.5 | 2.36 | 7.30 |
| 4.5 | 32.45 | 1.49 | 6.46 | 32.41 | 1.50 | 6.45 | 32.74 | 1.40 | 7.34 |
| 5 | 33.55 | 1.16 | 6.78 | 33.42 | 1.20 | 6.75 | 33.59 | 1.15 | 7.45 |

*Log DNA (dead cells) was derived through the subtraction of the log DNA value (post-drug exposure) for cells exposed to a specific concentration of ML from the log DNA value (post-drug exposure) for cells not subjected to ML.

**Figure S3**: : Dose-Response Profiling of Miltefosine on Intracellular Amastigotes of *Leishmania tropica* LS-LT2 (round of selection 5). The curves were generated by plotting the log DNA (dead cells) in relation to the concentrations of the drug.

**Table S8**: Dose-Response Profiling of Miltefosine on Intracellular Amastigotes of *Leishmania tropica* LS-LT2 (round of selection 10). This experiment was repeated three times in triplicate.

|  | Amastigotes (test samples - Replicate 1) | | | Amastigotes (test samples - Replicate 2) | | | Amastigotes (test samples - Replicate 3) | | |
| --- | --- | --- | --- | --- | --- | --- | --- | --- | --- |
| ML concentration (uM) | Ct | Log DNA (post-drug exposure) | Log DNA (dead cells)* | Ct | Log DNA (post-drug exposure) | Log DNA (dead cells) | Ct | Log DNA (post-drug exposure) | Log DNA (dead cells) |
| 0 | 10.72 | 7.93 | 0.00 | 10.72 | 7.93 | 0.00 | 10.71 | 7.94 | 0.00 |
| 1 | 11.31 | 7.76 | 0.18 | 11.3 | 7.76 | 0.17 | 11.31 | 7.76 | 0.18 |
| 2 | 12.28 | 7.47 | 0.46 | 12.35 | 7.45 | 0.48 | 12.42 | 7.43 | 0.50 |
| 3 | 14.47 | 6.82 | 1.11 | 14.46 | 6.82 | 1.11 | 14.59 | 6.79 | 1.15 |
| 4 | 15.81 | 6.42 | 1.51 | 15.89 | 6.40 | 1.53 | 16.02 | 6.36 | 1.57 |
| 5 | 17.51 | 5.92 | 2.01 | 17.66 | 5.88 | 2.06 | 17.89 | 5.81 | 2.13 |
| 6 | 18.73 | 5.56 | 2.38 | 19.04 | 5.47 | 2.47 | 19.24 | 5.41 | 2.53 |
| 7 | 21.37 | 4.77 | 3.16 | 21.68 | 4.68 | 3.25 | 21.84 | 4.63 | 3.30 |
| 8 | 25.24 | 3.63 | 4.31 | 25.36 | 3.59 | 4.34 | 25.67 | 3.50 | 4.44 |
| 9 | 29.78 | 2.28 | 5.66 | 29.91 | 2.24 | 5.69 | 30.12 | 2.18 | 5.76 |
| 10 | 33.11 | 1.29 | 6.64 | 33.29 | 1.24 | 6.70 | 33.42 | 1.20 | 6.74 |
| 12 | 35.09 | 0.70 | 7.23 | 35.34 | 0.63 | 7.31 | 35.68 | 0.53 | 7.41 |

*Log DNA (dead cells) was derived through the subtraction of the log DNA value (post-drug exposure) for cells exposed to a specific concentration of ML from the log DNA value (post-drug exposure) for cells not subjected to ML.

**Figure S4**: : Dose-Response Profiling of Miltefosine on Intracellular Amastigotes of *Leishmania tropica* LS-LT2 (round of selection 10). The curves were generated by plotting the log DNA (dead cells) in relation to the concentrations of the drug.

**Table S9**: Dose-Response Profiling of Miltefosine on Intracellular Amastigotes of *Leishmania tropica* LS-LT2 (round of selection 15). This experiment was repeated three times in triplicate.

|  | Amastigotes (test samples - Replicate 1) | | | Amastigotes (test samples - Replicate 2) | | | Amastigotes (test samples - Replicate 3) | | |
| --- | --- | --- | --- | --- | --- | --- | --- | --- | --- |
| ML concentration (uM) | Ct | Log DNA (post-drug exposure) | Log DNA (dead cells)* | Ct | Log DNA (post-drug exposure) | Log DNA (dead cells) | Ct | Log DNA (post-drug exposure) | Log DNA (dead cells) |
| 0 | 10.7 | 7.94 | 0.00 | 10.7 | 7.94 | 0.00 | 10.71 | 7.94 | 0.00 |
| 1 | 10.8 | 7.91 | 0.03 | 10.78 | 7.92 | 0.02 | 10.74 | 7.93 | 0.01 |
| 3 | 11.57 | 7.68 | 0.26 | 11.53 | 7.69 | 0.25 | 11.54 | 7.69 | 0.25 |
| 5 | 13.37 | 7.15 | 0.79 | 13.59 | 7.08 | 0.86 | 13.67 | 7.06 | 0.88 |
| 7 | 14.21 | 6.90 | 1.04 | 14.21 | 6.90 | 1.04 | 14.25 | 6.89 | 1.05 |
| 10 | 16.69 | 6.16 | 1.78 | 16.81 | 6.13 | 1.81 | 17.1 | 6.04 | 1.90 |
| 13 | 18.99 | 5.48 | 2.46 | 19.09 | 5.45 | 2.49 | 18.2 | 5.71 | 2.23 |
| 15 | 20.57 | 5.01 | 2.93 | 20.86 | 4.93 | 3.02 | 20.85 | 4.93 | 3.01 |
| 17 | 22.21 | 4.52 | 3.42 | 22.06 | 4.57 | 3.37 | 22.01 | 4.58 | 3.36 |
| 20 | 25.62 | 3.51 | 4.43 | 25.73 | 3.48 | 4.46 | 25.74 | 3.48 | 4.46 |
| 22 | 29.62 | 2.33 | 5.61 | 30.11 | 2.18 | 5.76 | 30.16 | 2.17 | 5.77 |
| 25 | 31.66 | 1.72 | 6.22 | 31.84 | 1.67 | 6.27 | 31.87 | 1.66 | 6.28 |

*Log DNA (dead cells) was derived through the subtraction of the log DNA value (post-drug exposure) for cells exposed to a specific concentration of ML from the log DNA value (post-drug exposure) for cells not subjected to ML.

**Figure S5**: : Dose-Response Profiling of Miltefosine on Intracellular Amastigotes of *Leishmania tropica* LS-LT2 (round of selection 15). The curves were generated by plotting the log DNA (dead cells) in relation to the concentrations of the drug.

**Figure S6**: 2D structure of beavericin


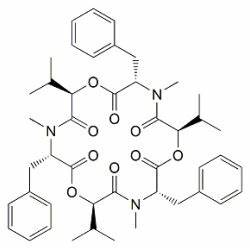


**Figure S7**: 2D structure of miltefosine


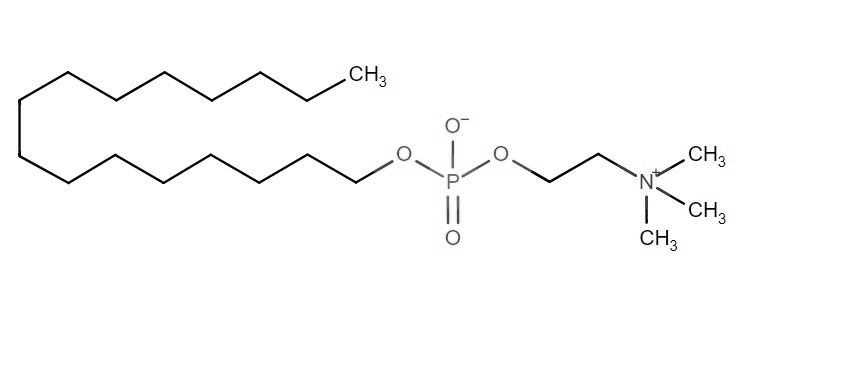

Supplement: Supplemental material — Tables S1 to S9, Figures S1 to S7, and Fasta sequence of the amplicons generated by the primers designed in this study. [file aac.01368-23-s0001.docx]
